# Interpretation of Kelvin Probe Force Measurements in Solid-State Electrochemical Cells

**DOI:** 10.1021/acsami.5c10182

**Published:** 2025-10-07

**Authors:** Franjo Weber, Chao Zhu, Shigeru Kobayashi, Till Fuchs, Taro Hitosugi, Jürgen Janek, Rüdiger Berger

**Affiliations:** † 28308 Max Planck Institute for Polymer Research, Ackermannweg 10, 55128 Mainz, Germany; ‡ Department of Chemistry, 13143 The University of Tokyo, Tokyo 113-0033, Japan; § Institute of Experimental Physics I and Center for Materials Research, 9175 Justus Liebig University Giessen, Heinrich-Buff Ring 17, 35392 Giessen, Germany

**Keywords:** Kelvin probe force microscopy, solid state electrolyte, mixed ionic electronic conductors, electrochemical potential, in operando

## Abstract

Kelvin probe force microscopy (KPFM) provides an established and reliable measurement of the work function of electronic conductors under equilibrium conditions. A less used but highly versatile application of KPFM is the characterization of electrochemical devices in operation, i.e., devices under nonequilibrium conditions. We derive the KPFM signal interpretation from basic considerations of the Volta potential and its relation to the surface potential, chemical potential of electrons, Galvani potential, and work function. As a key experiment for understanding operando measurements at electrochemical cells, we investigate a Hebb–Wagner solid-state polarization cell (HWC), constructed with a mixed ionic-electronic conductor (MIEC). Using a model-type MIEC based on amorphous Li_3_PO_4_, we illustrate how different potentials used in electrochemistry contribute to the KPFM signal. We show that KPFM measurements correspond to the inner electric (Galvani) potential profile along the HWC, once specific assumptions are valid. Consequently, KPFM can be very valuable in the investigation of solid electrolytes in operating electrochemical cells. Such cells are suitable models for all-solid-state batteries, candidates for future high energy density batteries.

## Graphical abstract

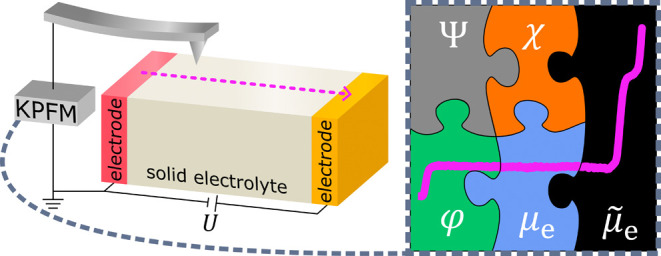

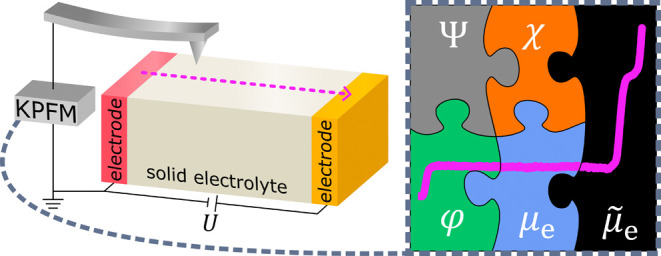

## 1 Introduction

A quantitative understanding of electrochemical cells, i.e., the charge transport and charge transfer steps therein requires advanced electrical measurements. One major approach is to use electrochemical impedance spectroscopy (EIS), which often helps to deconvolute major contributions to the resistive and capacitive properties of electrochemical cells.^1–3^ EIS is powerful and can be used under operando conditions. However, EIS is mostly applied macroscopically and does then not provide spatially resolved information. The impedance measured for heterogeneous materials can often only be reliably interpreted if detailed microstructure information is available. Furthermore, the impedance of full electrochemical cells is often hard to deconvolute due to overlap of anode and cathode contributions. This calls for impedance measurements with 3-electrode cells that require advanced cell setups. While advanced EIS interpretation based on distribution of relaxation times recently helps to deconvolute complex spectra better, the resulting equivalent circuit-based interpretations are not unequivocal. Local impedance measurements with miniaturized electrodes have been frequently reported, but are by no means a routine technique.^4^ Here the need for well contacted electrodes with a defined contact geometry seriously limits this approach. For this reason, mechanically contact-free techniques recently attract increasing interest. Among these, Kelvin probe force microscopy (KPFM) appears to be a versatile technique, that is already widely used in other fields than electrochemistry, e.g., in photovoltaics,^5,6^ electronics^7^ and optics.^8^ Nowadays, KPFM modes are routinely available in commercial instruments which allow to perform KPFM measurements—yet the interpretation of the data is often unclear.

In KPFM, the electrostatic potential difference between a sharp, electrically conductive tip and a sample surface is measured. This potential difference causes an electrostatic force that acts locally between the tip apex and the sample surface. The electrostatic force is minimized by applying a compensating voltage *U* between the tip and the sample. In KPFM, this voltage *U* is mapped locally. A lateral resolution down to subnanometers is possible.^9^ For electronically conductive samples the measured voltage *U* coincides with the contact potential difference (*U*_CPD_) that is the key experimental observable in KPFM.^9^ KPFM is widely applied to map variations in surface composition,^10^ to investigate charge separation upon illumination,^11^ to detect electronic and/or ionic charge movement inside electronic devices,^12^ and to detect charges deposited on insulating surfaces.^13^ Furthermore, the materials, the types of samples and their applications being studied are quite diverse. Particularly, samples with mobile ionic charge carriers—in addition to electronic charge carriers—provide unusual conditions compared to samples with exclusively electronic conduction, like semiconductors and metals. Electrochemical cells rely on components that are either purely ion-conducting or mixed ion-/electron-conducting. Therefore, local composition changes during a measurement can compromise simple interpretations transferred from purely electronic devices. Thus, the question arises how the measured contact potential difference is correctly interpreted in each individual case.

In this paper, we focus on the signal interpretation of *U*_CPD_ but do not go into the various implementations and technical tricks for realizing KPFM. The first purpose is to provide an overview of various KPFM scenarios ranging from ″equilibrium measurements″ to ″operando measurements″ for electronic but also ionic conductors and to insulators. Second, we like to explicitly clarify the signal interpretation for samples with mobile ionic charge carriers. The third purpose is to reduce language barriers that clearly exist between the typical physical nomenclature of metal and semiconductor physics and the typical electrochemical nomenclature. Different nomenclatures^9,14^ are used to describe the results of measurements, depending on the field of science—which clearly hampers understanding of KPFM measurements and a widespread use of KPFM. The inconsistency of terms used by either physicists or electrochemists was already noted by E. Lange^15^ for the general case and highlighted by Örnek et al.^16^ for the specific case of KPFM. However, a consideration of operating devices concerning the different terminologies is still missing. This unintended language barrier can be confusing and even leads to controversial discussion. For the above reasons, we aim to pave the way toward a common understanding of the signal interpretation of KPFM on devices in operation, regardless of the specific research background.

To provide an overview of signal interpretation, we first review KPFM experiments from the literature and categorize them into “equilibrium measurements” and “operando measurements” (i.e., nonequilibrium measurements). We then introduce the terminology used in electrochemistry by reviewing the electrochemical potential of electrons. Based on this terminology, we outline the theoretical basis of KPFM. By studying a model system of a MIEC under *operando* conditions, we explain how different potentials known from electrochemistry contribute to the KPFM signal. Finally, we present signal interpretation for specific sample boundary conditions.

## 2 KPFM Signal Basics

For an electronically conductive sample in (electro)­chemical equilibrium, the *Fermi level* is constant in space and time. Electrochemists say that the *electrochemical potential* of electrons ( μ̃ _e_) is constant, i.e., gradient free. The *U*_CPD_ in an equilibrium measurement (Figure 1a) is then equal to the difference between the work function of the tip ϕ_t_ and sample ϕ_s_ divided by the Faraday constant *F*, eq 1:[Fn fn1]

1 $$U_{\mathrm{CPD}}=\frac{\phi_{s}-\phi_{t}}{-F}=\frac{\Delta \phi }{-F}$$

**Figure 1:**
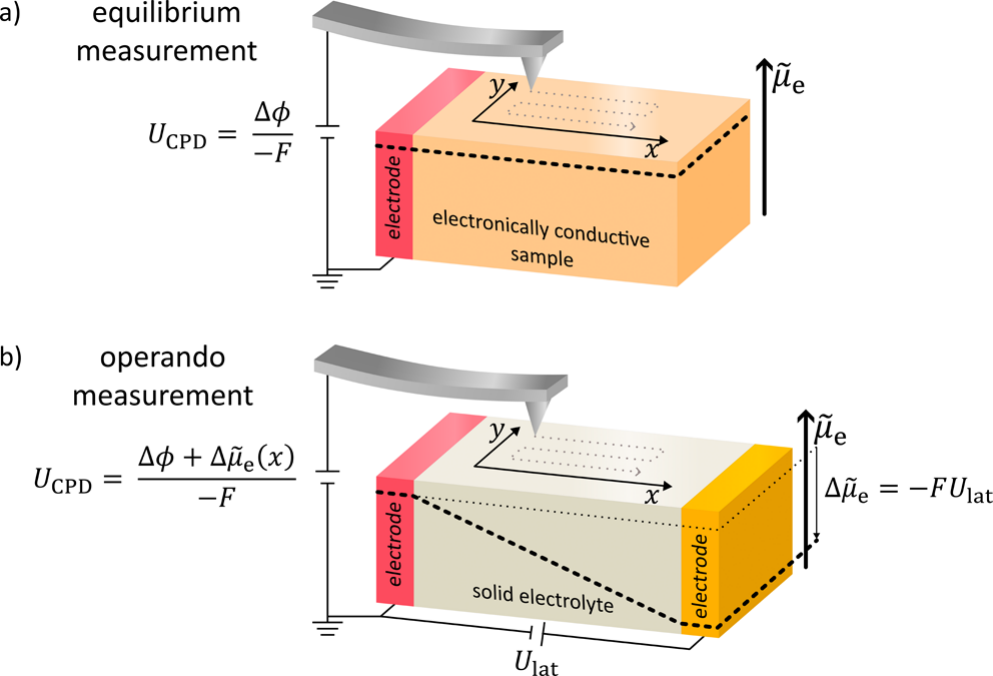
Typically, KPFM is performed on samples in electrochemical equilibrium, as shown in (a). The sample (here an electronically conducting material) can be electrically connected from either side—here shown from the left side. The electrochemical potential of electrons has the same value everywhere, which we illustrate by the dashed line. The signal interpretation for this case is well known.^9^ (b) For an operando measurement, the signal interpretation is more sophisticated. The sketch shows an example of such a measurement, where KPFM is performed on a solid electrolyte in an all-solid-state battery. Due to the lateral voltage *U*_lat_ between the two electrodes, the electrochemical potential of electrons μ̃ _e_(*x*) is position dependent (dashed line, here a gradient exclusively in one dimension is assumed).

In case the work function of the tip ϕ_t_ is known, KPFM allows a quantitative measurement of the work function of the sample surface ϕ_s_(*x*,*y*) as a function of position.^18^ Based on this assumption, Sharma et al. reported that nano-roughened, sol–gel-derived polycrystalline ZnO thin films show coexistence of two work function values on a submicrometer scale, possibly due to heterogeneous crystallinity or crystal orientation. The work functions measured by KPFM were quantitatively consistent with work functions measured by ultraviolet photoelectron spectroscopy (UPS) for the same material.^19^

Furthermore, the quantitative measurement of the work function allows the analysis of electrochemical processes. In this way, Ma et al. calibrated the measured potential by KPFM with the hydrogen concentration on a nickel surface. The authors found a linear relationship between the measured Volta potential and the hydrogen concentration.^20^

A much less explored application of KPFM is the characterization of devices in operation. ’In operation’ means that charge carriers move from one position to another due to an external stimulus. Then the system is not in electrochemical equilibrium and the Fermi level (electrochemical potential of electrons μ̃ _e_) along the cell varies spatially, depending on the local charge transport and transfer conditions. Studies of devices with KPFM in operation are highly valuable, as the lateral resolution is superior, a sub-millisecond temporal resolution can be achieved and information on the behavior of individual components of functional devices can be obtained. In a recent study, Weber et al. performed *operando* KPFM measurements on cross sections of perovskite solar cells to map the redistribution of ionic charges upon a light or voltage pulse.^6^ Qi et al. recently reviewed how KPFM can be utilized to obtain information in solid state batteries, where the solid electrolyte is mainly conductive for ions and the electronic conductivity is low. The authors emphasized that *operando* KPFM can be used to analyze interfacial potential drops in dependence of cell voltage.^21^ Fueller et al. showed that a large drop in the interfacial potential is associated with a rate-limiting step.^22^ Sometimes a cross section of the device, e.g., a solid state battery cell, is fabricated for *operando* KPFM. KPFM is then performed on top of the cross section while the device is operated externally,^21–24^ i.e., by applying a lateral voltage *U*_lat_ (Figure 1b). This lateral voltage causes a gradient in the electrochemical potential of electrons across the device (dashed line, Figure 1b). Eq 1 is now not valid anymore and has to be extended by a nonequilibrium term Δ μ̃ _e_(*x*) leading to eq 2. The nonequilibrium term denotes the difference in the electrochemical potential of electrons between the measured position and the ground.^21,22^

2 $$U_{\mathrm{CPD}}\left(x\right)=\frac{\Delta \phi +\Delta {\mathrm{\mu ̃}}_{\;e}\left(x\right)}{-F}$$

For the analysis of devices in operation, typically line profiles of *U*_CPD_(*x*) are recorded via KPFM (gray dotted lines, Figure 1). For the signal interpretation according to eq 2, *U*_CPD_(*x*) is measured, while Δϕ and Δ μ̃ _e_(*x*) are unknown. As Δ μ̃ _e_(*x*) is not easily accessible in most cases, the work function difference often cannot be calculated by eq 2. Instead, the *U*_CPD_(*x*) profile is then interpreted either as the profile of the potential acting inside the sample^25^ or as the profile of the potential acting on the surface of the device.^26^ In the latter case, surface band bending contributes to the measured potential profile as well. Here, we aim to clarify the signal interpretation for devices in operation. Specifically, we derive a signal interpretation of *operando* KPFM measurements along electrochemical cells for solid electrolytes.

## 3 Electrochemical Potential of Electrons

The electrochemical potential of electrons plays a key role, as it is the quantity that connects the energy landscape that electrons experience in an electrochemical cell to the electronic “environment”, i.e., to the electric circuitry connected to the cell.

Consider an electronic conductor surrounded by vacuum. We are interested in the work required to transfer an electron with an initial energy of zero into our sample, which is an electron conductor. Inside the conductor, the electron has an energy which corresponds to the electrochemical potential of electrons μ̃ _e_. The starting point of the thought experiment is given by the electron, at infinite distance from all other charges and the electronic conductor. There at position **R**, by definition, the test charge has a potential energy of zero (Figure 2a). Then the test charge is transferred into the sample. The first step corresponds to the Volta potential. The **Volta potential Ψ** is defined as the electrostatic potential difference between **R** and a point **S** “just above” (≈10 nm) the surface (gray arrow, Figure 2a).^16^ When the sample is charged, an electrostatic field determines the work required to move the test charge from the point **R** to **S**.^16^ At shorter distances to the surface, dispersion interactions play a role. The interaction with induced image charges has the longest range (red arrows, Figure 2a).^27^ Therefore, dispersion forces with the test charge at distances <10 nm cause an additional potential contribution.^27^ For this reason, the Volta potential is defined only to the point **S**, which is close enough to capture all contributions from the excess charge of the sample, but far away enough that image charges can be neglected.^29^ Additional work is required to move the test charge from point **S** to a point **I** within the sample (Figure 2b). The electrostatic part of this work is associated with the **surface potential** ***χ*** and arises from the electric double (dipole) layer at the surface (orange arrow, Figure 2b).^16^ The dipole layer depicted for a metal surface (blue and red partial charges, Figure 2b) generally exists at all surfaces.^30^ The sum of the Volta potential and the surface potential is defined as the **Galvani potential** ***φ***, which represents the electrostatic potential acting inside the sample (green level, Figure 2b).

3 φ=Ψ+χ

**Figure 2:**
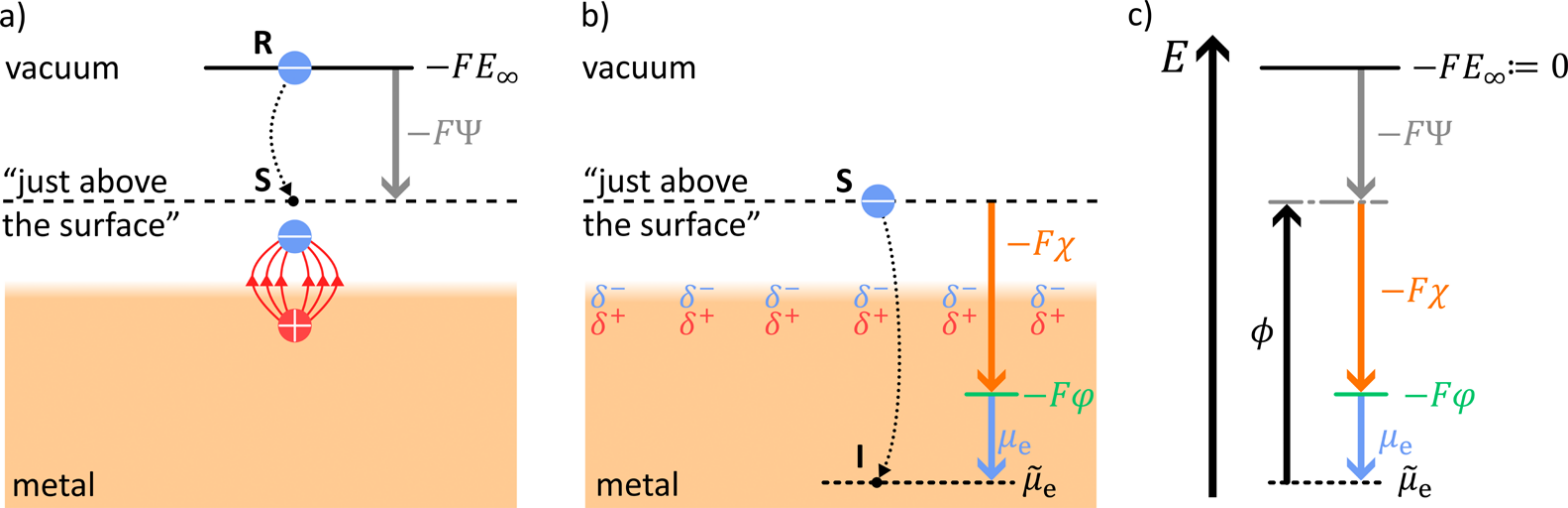
(a) The reference point **R** is chosen at infinite distance from all charges and has an electrostatic potential of *E*_∞_ = 0. The work required to move an electron from the reference point **R** to a point **S** “just above” the surface is associated with the Volta potential Ψ. If the electron is moved closer than “just above the surface”, interactions with image charges will no longer be negligible.^27^ (b) The work required to move an electron from the point **S** to a point **I** within the sample is associated with the surface potential χ and the chemical potential of electrons μ_e_.^27^ (see SI1 for consideration of sign). (c) Schematic representation of potentials in an energy diagram.^28^

The Galvani potential φ is often also named as inner electric potential or simply as electric potential.^31^ It is the electric potential that plays the essential role in discussing all electrochemical phenomena within condensed phases. Finally, there is an additional work contribution related to the chemical interactions in which the electron takes part inside the sample. This work is named **chemical potential of electrons** ***μ***_**e**_ (blue arrow, Figure 2b).^29^

The total amount of work required to transfer an electron from **R** to point **I** is the sum of the above potentials and represents the **electrochemical potential of electrons** ***μ̃***_**e**_ (Figure 2c). In general, the electrochemical potential of any charged particle j can thus be described by eq 4.

4 $${\mathrm{\mu ̃}}_{j}=\mu_{j}+z_{j}F\left(\chi +\Psi \right)$$

Here, *z*_j_ is the charge number of the particle j in units of the elementary charge and *F* is the Faraday constant. For example, in case j = e, we consider the electrochemical potential of electrons, thus *z*_e_ = −1. We note that the separation of the work required to move an electron from point S to point I is only conceptually. In practice, the chemical potential of electrons μ_e_ and the surface potential χ cannot be measured separately, as we cannot separate the electron from its charge. One can measure the energy required to remove an electron from the sample (point I) to a point just above the surface (point S). This energy is called work function ***ϕ*** (eq 5, Figure 2c):^32^

5 $$\phi =-\mu_{\;e}+F\chi$$

The work function is a material specific property and thereby characteristic for the respective composition of a sample and surface orientation in case of crystalline domains. The work function is defined starting from the energy level of the electrochemical potential of electrons μ̃ _e_ and points in the opposite direction of μ_e_ and χ.^27^ The different physical meanings of the work function and the (negative) electrochemical potential of electrons become obvious when we consider a metal sample with different crystalline facets. In equilibrium, the electrochemical potential of electrons is homogeneous throughout the sample, i.e., the Fermi level is equal everywhere. Yet, different crystal facets of the same metal have different work functions. This implies that the Volta potential is necessarily different above the different facets—which would be detected by the Kelvin probe, see next section.

## 4 Basic Principle of KPFM

Typically, ϕ is measured by ultraviolet photoelectron spectroscopy, however, with a relatively low spatial resolution. For a more detailed description of this technique the reader is referred to the work of Cahen et al.^33^ Spatial variations in ϕ can be measured with nanometer resolution by KPFM,^18^ which we discuss next for electronically conductive sample surfaces (Figures 3–5).

**Figure 3:**
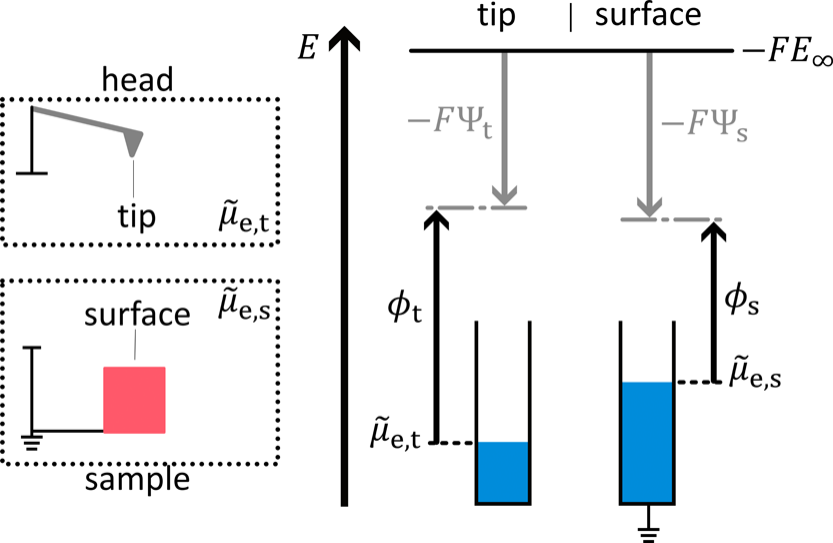
Operating principle and signal interpretation of KPFM. The dotted frames indicate regions with the same electrochemical potential of electrons. We distinguish between the KPFM head and the sample. The electrochemical potential of electrons equilibrates within head and sample if they are not electrically connected. KPFM head and sample are often heterogeneous. Therefore, the Volta potential can vary locally over these regions. The energy diagram refers to the tip of the KPFM head and the surface of the sample, as these spots are the most relevant to the measurement. The electrochemical potential of electrons for the tip is μ̃ _e,t_, and the corresponding Volta potential is Ψ_t_. The same parameters with index s apply to the sample surface.

**Figure 4:**
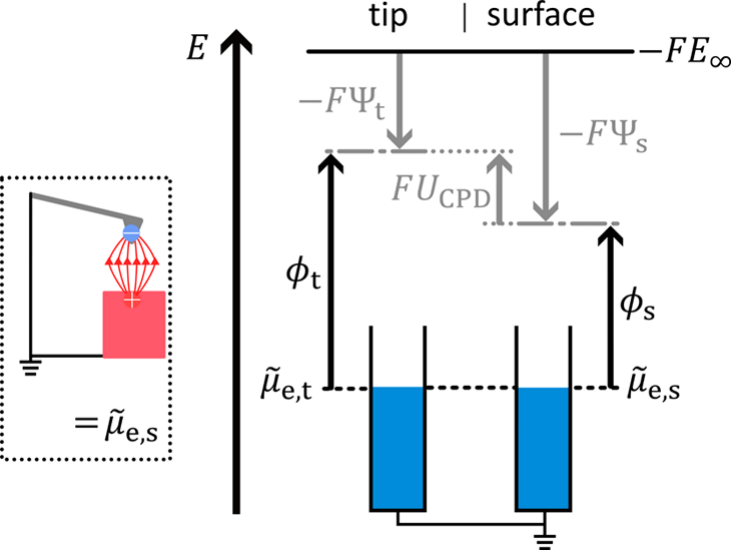
An electronic connection (short circuit) between head and sample is established. The latter leads to an equilibration μ̃ _e,t_= μ̃ _e,s_. The different work functions cause a Volta potential difference (*U*_CPD_).

If the KPFM head is not electronically connected to the sample, the electrochemical potential of electrons will only equilibrate within the corresponding regions, i.e., μ̃ _e,t_ for the head and μ̃ _e,s_ for the sample, respectively (Figure 3). Consequently, the tip has a specific Volta potential Ψ_t_ and the surface of the sample another Ψ_s_.

Once the head and sample are electrically connected (short-circuit), electrons flow in the direction of the lower μ̃ _e_. The current disappears when the electrochemical potential of electrons is balanced, i.e., μ̃ _e,t_ = μ̃ _e,s_ (Figure 4). The latter results in the buildup of opposing charges at the tip and surface, causing the Volta potentials to change accordingly. The Volta potential difference ΔΨ is called contact potential difference

6 $$\Delta \Psi =U_{\mathrm{CPD}}=\Psi_{s}-\Psi_{t}$$

*U*_CPD_ can also be expressed by the work functions of the tip and sample (black arrows, Figure 4). Using the condition for a balanced electrochemical potential of electrons ( μ̃ _e,t_ – μ̃ _e,s_ = 0) and rewriting μ̃ _e_ by the sum of the Volta term minus the work function, −*FΨ* – ϕ for the tip and the sample respectively, we obtain eq 7.

7 $$U_{\mathrm{CPD}}=\Psi_{s}-\Psi_{t}=\underset{=0}{\underset{︸}{\frac{{\mathrm{\mu ̃}}_{\;e,t}-{\mathrm{\mu ̃}}_{\;e,s}}{F}}}+\Psi_{s}-\Psi_{t}=\frac{\phi_{s}-\phi_{t}}{-F}$$

Importantly, ΔΨ between the tip and the surface causes an electrostatic force. In a simple approach, the electrostatic force can be calculated by treating the electrostatic tip–surface system as a capacitor

8 $$F_{\mathrm{el}}=\frac{1}{2}\frac{dC}{dd}{\left(\Delta \Psi \right)}^{2}$$

with the capacitance denoted as *C* and the distance between tip and sample as *d*.^34^ KPFM detects the electrostatic force and thus is a noncontact technique for mapping the *U*_CPD_ (ΔΨ). Particularly, KPFM measures the contact potential difference by applying a direct current (DC) voltage *U*_DC_ between KPFM head and sample. The magnitude of *U*_DC_ is adjusted by an electronic feedback loop such that the electrostatic force between tip and surface is minimized. The tip is connected to the positive pole and the sample surface to the negative pole of the voltage source (Figure 5). To compensate a positive *U*_CPD_, a positive *U*_DC_ must be applied between the poles. The electrostatic force is minimal when the Volta potential difference between tip and surface disappears (eq 8). Then *U*_DC_ = *U*_CPD_ (see SI1 for a consideration of sign). For a more detailed explanation of the technical implementation of different KPFM modes, the reader is referred to the work of Axt et al.^34^

**Figure 5:**
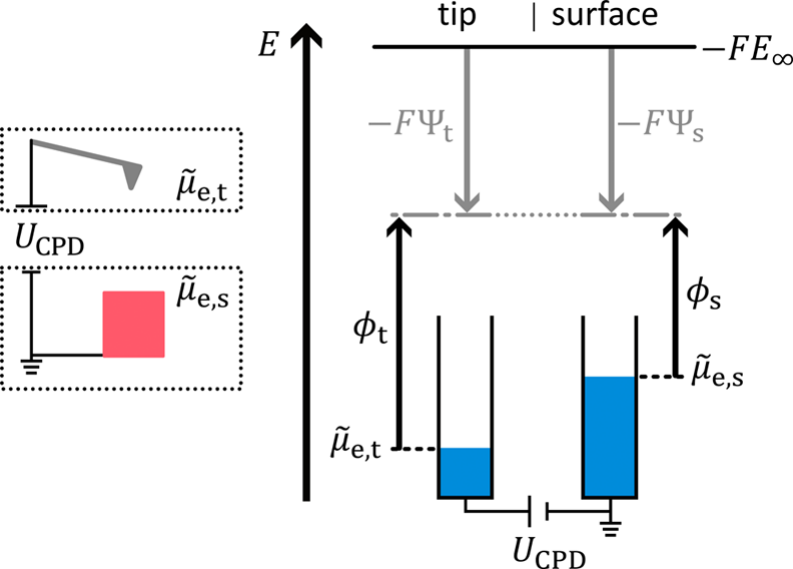
KPFM in operation, i.e., a compensating voltage is applied, that equals the work function difference (see SI1 for consideration of sign). Figures 3–5 are adapted from reference 16.

The above analysis assumes an electronically conductive sample. For insulating samples, the electrochemical potential of electrons can only equilibrate for the KPFM head and the electrically connected sample back electrode of the insulator (dotted frame, Figure 6a). Thus, if charges *q*_*i*_ are present at positions ***r***_*i*_, they will cause an additional electrostatic potential distribution. The Volta potential difference measured by KPFM can be represented by eq 9.

9 $$\Delta \Psi =U_{\mathrm{CPD}}+\underset{i}{\sum }q_{i}W\left(r_{i}\right)$$

Here, *W*(***r***_*i*_) is the weight function. The weight function is calculated for each charge and describes its contribution to the electrostatic potential for a given measurement position.^35^ We emphasize that here the *U*_CPD_ reflects electrical connections to the sample back electrode and to the particular KPFM setup. Therefore, the measured ΔΨ values vary with different electrode materials, KPFM hardware (e.g., head and tip holder) and KPFM-tip coatings. During KPFM, an electrostatic force is generated between tip and back electrode to compensate electrostatic forces resulting from localized charges. The forces are balanced if the potential energy of the localized charges (*E*_*l*_) and the negative potential energy inside the tip-back electrode capacitor (−*E*_*C*_) have the same slope at a height *h* above the surface (red dotted line, Figure 6b). The potential energy inside a capacitor decreases linearly with increasing thickness *d*. Consequently, for a thicker insulator (*d*_2_ > *d*_1_), a larger voltage (*U*_2_ > *U*_1_) is required to compensate the electrostatic forces at height *h* above the surface. Thus, the thickness of the insulator plays a crucial role when measuring the potential of localized charges. With increasing thickness of the insulator, the applied potential between tip and back electrode to compensate the electric field emanating from the localized charges rises. The voltage limit of commercial KPFM instruments is often 10 V, set by the maximum bias supply. Therefore, with a surface charge density as little as 2 nC/cm^2^ an insulating layer like, e.g., quartz should be thinner than 20 μm to ensure stable KPFM measurements.^36^ Zhou et al.^13^ analyzed the distribution of localized charges via KPFM after nanoscale triboelectrification and Knorr et al.^36^ measured the surface charge density left behind on polymer surfaces after being dewetted by water.

**Figure 6:**
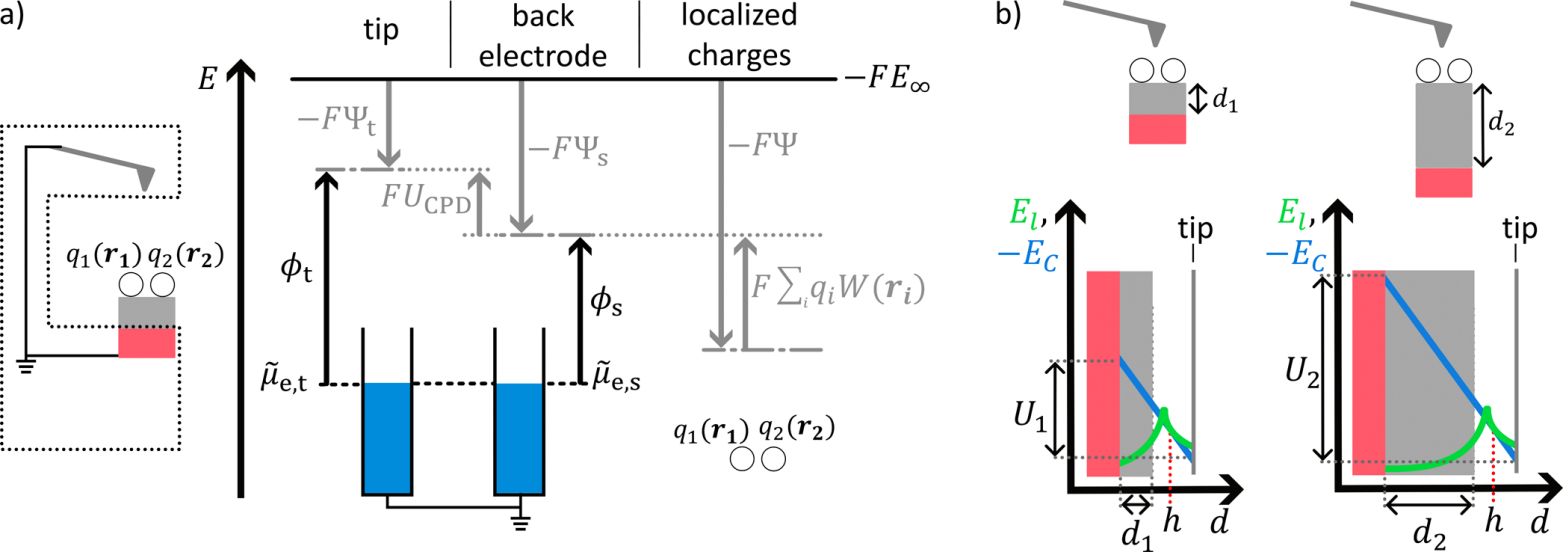
(a) Operating principle and signal interpretation of KPFM on an insulator (gray) with deposited charges *q*_*i*_ at position ***r***_*i*_ on top of a back electrode (red). The KPFM setup is shown in the short-circuited case to separate the contributions of the Volta potential difference between tip and surface. The dotted frames indicate regions with the same electrochemical potential of electrons. The acting Volta potential difference between sample and tip has a contribution of the contact potential difference between tip and back electrode and an electrostatic contribution emerging from the localized charges. Adapted from references 16 and 37 . (b) Change in applied potential for thin (left) and thick (right) insulators. During KPFM, an electrostatic force is generated between tip and back electrode to compensate electrostatic forces resulting from localized charges. The forces are balanced if the potential energy of the localized charges (*E*_*l*_) and the negative potential energy inside the tip-back electrode capacitor (−*E*_*C*_) have the same slope at a height *h* above the surface (red dotted line). The potential energy inside a capacitor decreases linearly with increasing thickness *d*. Consequently, for a thicker insulator (*d*_2_ > *d*_1_), a larger voltage (*U*_2_ > *U*_1_) is required to compensate the electrostatic forces.

### Operando KPFM Applied to Full Electrochemical Devices

We discussed the formalism for the KPFM measurement of samples that are electronically conductive and in electrochemical equilibrium, i.e., μ̃ _e_ is constant in the sample (Figure 1a). Operando measurements often require to alter the electrochemical potential of electrons within the sample (Figure 1b). First, we consider two identical metals as electrodes. The left metal electrode is grounded and we apply a positive voltage *U*_lat_ vs ground to the right electrode (Figure 7a). The electrochemical potential of electrons in the right electrode shifts from its original value μ̃ _e,s_ to μ̃ _e,s’_ = μ̃ _e,s_ – *FU*_lat_ (see SI1 for a consideration of sign). The work function of the metal remains constant as long as the chemical composition stays unchanged.^28^ So the Volta potential shifts from its original value Ψ_s_ to Ψ_s′_ = Ψ_s_ – ( μ̃ _e,s′_ – μ̃ _e,s_)/*F*. Substituting this into eq 6 we get

10 $$U_{\mathrm{CPD}}=\Psi_{s\prime }-\Psi_{t}=\underset{=0}{\underset{︸}{\frac{{\mathrm{\mu ̃}}_{e,t^{\prime }}-{\mathrm{\mu ̃}}_{e,s^{\prime }}}{F}}}+\Psi_{s}-\Psi_{t}-\frac{\left({\mathrm{\mu ̃}}_{e,s^{\prime }}-{\mathrm{\mu ̃}}_{e,s}\right)}{F}=\frac{\phi_{s}-\phi_{t}}{-F}+U_{\mathrm{lat}}$$

**Figure 7:**
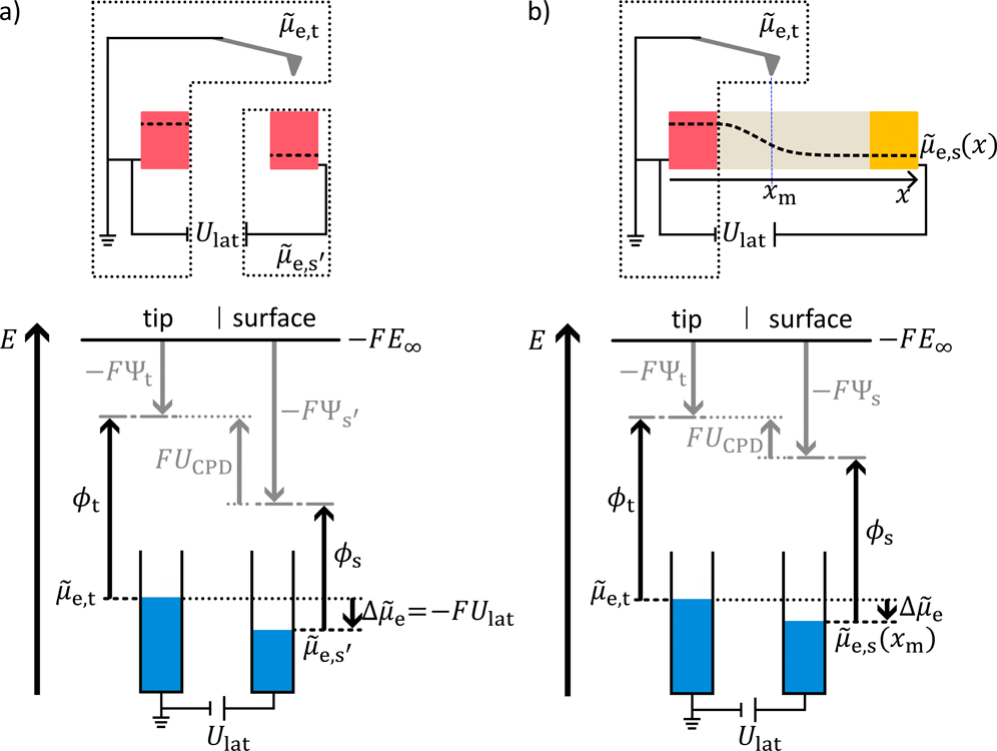
Operating principle and signal interpretation of KPFM on samples with an externally applied lateral voltage. The KPFM setup is shown in the short-circuited case. (a) Electronic connection between KPFM head and sample with an applied voltage *U*_lat_. (b) Electronic connection between KPFM head and a laterally polarized sample. The electrochemical potential of electrons in the sample μ̃ _e,s_(*x*) depends on the lateral position *x*.

Therefore, applying an additional voltage *U*_lat_ to the sample results in a shift of *U*_CPD_ by the local contribution of *U*_lat_. We like to note that this conclusion is only correct if the sample will not change its composition upon applying a bias. This assumption is fulfilled for a purely electron-conducting sample. However, for a mixed electron- and ion-conducting material the composition can change due to field-driven migration of ions. This migration depends on the type of MIEC and the specific electrode materials being part of the electrochemical cell. In this case the interpretation of the measured *U*_CPD_ is different, which we discuss in more detail below.

To illustrate operando KPFM measurements, we consider a device in which an electron-conducting material is sandwiched between two electrodes. The device is operated by applying a voltage *U*_lat_ between the two electrodes. We analyze the scenario where the KPFM head is electrically connected to one electrode of the sample (Figure 7b). For devices in operation, there is a gradient in the electrochemical potential of electrons in the sample ( μ̃ _e,s_(*x*)) owing to an external stimulus, which is here *U*_lat_. Then the sample is no longer in electrochemical equilibrium (Figure 1b). Here we assumed that all phases in the device are electronically conductive. Then Δ μ̃ _e_ is not constant between the tip and each measurement point (*x*_m_) on the sample, owing to the acting internal changes of the electrochemical potential of electrons (dashed black line, top of Figure 7b). In addition, ϕ_s_ is unknown owing to compositional changes within the sample. Thus, eq 2 cannot be used to interpret the measured *U*_CPD_. For a signal interpretation in this case, we rewrite the expression for *U*_CPD_ (eq 6) using the definition of the Galvani potential (eq 3) and obtain eq 11.

11 $$U_{\mathrm{CPD}}=\Psi_{s}-\Psi_{t}=\left(\varphi_{s}-\chi_{s}\right)-\Psi_{t}$$

Then, we calculate the derivative d/d*x* =∇ along the *x* direction, which is the direction of the potential profiles recorded via KPFM, and take into account that Ψ_t_ remains constant during a measurement. We obtain

12 $$\nabla U_{\mathrm{CPD}}=\nabla \varphi_{s}-{\nabla \chi }_{s}$$

The measured potential profile depends on the spatial variation of the Galvani potential (∇φ_s_) and a possible variation of the surface potential (∇χ_s_). As mentioned above the surface potential results from the electrical double layer (dipole) of the surface and reflects the (chemical) structure of the surface. For systems with no significant compositional gradient, we can assume that the dipole at the surface is spatially homogeneous. Hence ∇χ_s_ = 0 and the measured ∇*U*_CPD_ corresponds to ∇φ_s_. Bürgi et al.^25^ used this signal interpretation to study thin-film field-effect effect transistors. They showed that KPFM maps the local electrostatic potential within the accumulation layer of a transistor on the nanometer scale. The electrostatic potential mentioned corresponds to the Galvani potential in electrochemical nomenclature. For samples where compositional gradients are no longer negligible, the surface dipole may get inhomogeneous leading to a contribution to the measured ∇*U*_CPD_ (Figure 8). The latter can indeed be the case: Saraf et al.^26^ compared cross-sectional KPFM measurements on silicon diodes with calculated bulk potential profiles. They reported differences between the electrostatic potential profiles at the surface and in the bulk due to concentration gradients of charged surface states.

**Figure 8:**
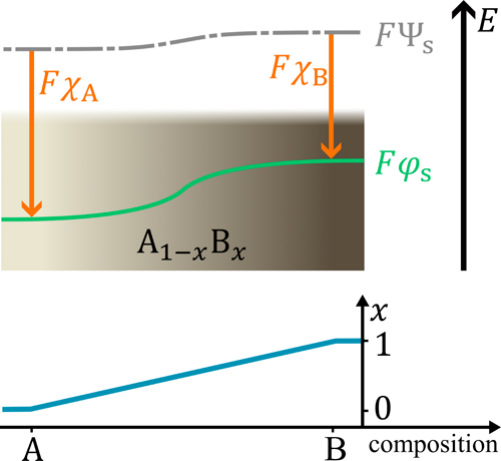
Sketch of a sample with a lateral compositional gradient. Surface potential gradients cause a difference between the Volta and Galvani potential profiles.

Next, as an example for electrochemically induced compositional gradients, we consider ionic conductors with a low electronic conductivity, i.e., solid electrolytes. These conductors belong to a class of materials known as mixed ionic-electronic conductors (MIECs). This terminology is equivalent to ’ionic semiconductors’ in physics, such as nonstoichiometric silver sulfide. However, designation as an MIEC is not restricted to a material with a specific type of bonding, but rather a classification that only accounts for the conduction properties of a sample.^38^ Most MIECs show nonstoichiometry to a certain extent, i.e., their composition within a single phase material can vary within the same (lattice) structure, as described by the width of the phase field in the corresponding phase diagram. Their use in different technologies depends on their specific conductivity properties, e.g., materials with equally high electronic and ionic conductivity are used as insertion- or intercalation-type electrode materials.^39^ MIECs with very high ionic conductivity and very low electronic conductivity are used, e.g., as solid electrolytes in the development of all-solid-state batteries that could enable high power and high energy density.^40^

## 5 Hebb–Wagner Cell

When laterally biased by a voltage *U*_lat_, MIECs in all-solid-state batteries show a nonuniform composition due to a lateral concentration gradient of minority charge carriers, i.e., electrons.^41^ An elegant way to separate the surface potential from the Galvani profile is the analysis of a so-called Hebb–Wagner cell^42,43^ (HWC).

The HWC consists of a reference electrode (RE, left side of cell) that is reversible for the ionic charge carrier and ideally provides a chemical potential reference, e.g., by using the parent metal M in case of mobile cations M^+^. The MIEC and an inert ion-blocking electrode that constitutes the working electrode (WE, right side of cell) complete the asymmetric polarization cell (Figure 9a). We assume that the MIEC is a good ion conductor with a low electronic conductivity. The two electrodes have different charge transfer properties at their interface with the MIEC. The RE allows reversible transfer of both ions and electrons, and it is the stable thermodynamic reference of the cell. The chemical potential of the metal as neutral component, as well as the electrochemical potential of ions and of electrons are fixed by this RE. The ion-blocking WE is reversible for electrons only, once it is polarized toward positive potentials (polarization direction)—as it cannot supply metal ions M^+^. As the potential difference between RE and WE is not caused by reversible chemical reactions at either electrode, the HWC does not qualify as a battery.

**Figure 9:**
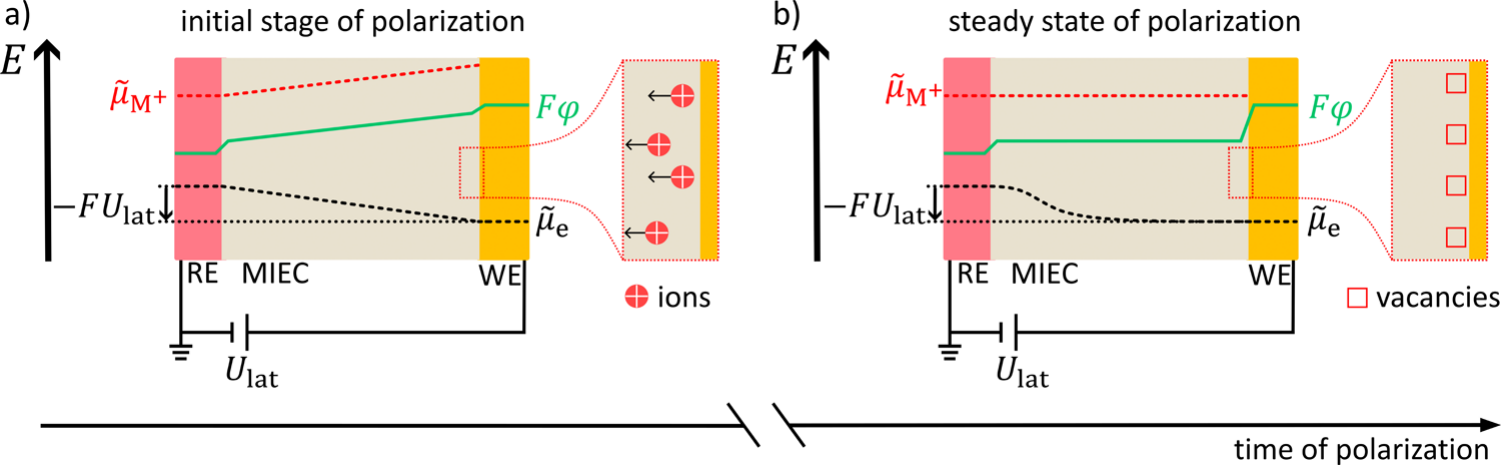
Scheme of the polarization process of a Hebb–Wagner cell by applying a voltage *U*_lat_. Cell setup from left to right: reversible reference electrode (RE), mixed ionic-electronic conductor (MIEC) with mobile cations, and an ion-blocking working electrode (WE). Profiles of the electrochemical potential of cations ( μ̃ _M+_), Galvani potential (φ), and electrochemical potential of electrons ( μ̃ _e_) are shown in the initial state of the polarization cell (a) and in the steady state (b) of polarization. During polarization, the gradient in the Galvani potential profile causes ions to migrate toward the reversible electrode (inset in (a)), where they are discharged. The movement of ions creates vacancies as part of the space charge layer toward the ion-blocking electrode that shield the MIEC from the electric field in the steady state (inset in (b)). Moreover, the movement of ions generates nonstoichiometry in the MIEC bulk, which causes a gradient in the electrochemical potential of electrons (dashed black line, (b)).

When a voltage *U*_lat_ is applied between RE and WE in polarization direction, the electric field emanating from the two electrodes propagates at the speed of light. Due to their finite conductivity, charge carriers cannot move as quickly as the electric field builds up. Thus, in the initial stage of polarization, we can treat the HWC with two electrodes as a capacitor, where the Galvani potential inside the MIEC increases linearly (green profile, Figure 9a). The chemical potential of metal ions μ_M+_ is constant within the MIEC in the pristine state. Consequently, the profile of the electrochemical potential of metal ions μ̃ _M+_ (eq 4) has the same slope inside the MIEC as the Galvani potential. A gradient in μ̃ _M+_ is the driving force for a current of metal ions. The metal ions are accelerated toward the reversible electrode (inset, Figure 9a). Due to the ion-blocking character of the WE, there is no ion supply from this electrode. Therefore, cation vacancies (with formally negative charge) gradually form in the MIEC, leading to a small metal deficiency extending from the WE into the bulk of the MIEC. These shield the electric field and lead to a decreasing gradient in the Galvani potential (inset, Figure 9b). As a result, the ion current diminishes and eventually disappears in the stationary state—if no decomposition takes place at the WE. The solid electrolyte-type MIEC is a good ionic conductor with a high concentration of mobile ions, as well as a very narrow phase field. Therefore, we can safely assume that the local changes in the concentration of mobile M^+^ ions due to shielding of the electric field are very small.^44^ Under this assumption, the chemical potential μ_M+_ of M^+^ ions is practically not altered and is gradient free within the MIEC.^45^ It follows from eq 4, that the electrochemical potential μ̃ _M+_ of M^+^ ions is also gradient free (red dashed line, Figure 9b), since both the profiles of φ and μ_M+_ in the MIEC are gradient free. In essence, a Hebb–Wagner polarization cell works like a capacitor with a perfectly polarizable dielectric.

Local charge neutrality must prevail in the MIEC bulk, and the net movement and “extraction” of the M^+^ ions from the MIEC bulk during polarization leads to a local charge imbalance that needs to be compensated by electrons or holes. Since these are minority charge carriers, their resulting concentration changes are not negligible and thus locally alter the chemical potential of electrons. The latter causes a gradient in the chemical potential of the electrons during polarization, resulting in a gradient in μ̃ _e_ (black dashed line, Figure 9b). This gradient causes a finite and constant electronic (diffusion) current that is only driven by a concentration gradient of electrons, not by an electric field.^44,46^

In short, a HWC allows to test the influence of the gradient of the electron/hole concentration on ∇*U*_CPD_, caused by a minor compositional change in the bulk of the MIEC. The Galvani potential profile in the MIEC is flat in the steady state; hence, ∇φ_s_ = 0. Thus, by measuring ∇*U*_CPD_, the surface potential gradient ∇χ_s_ can be determined.

## 6 Results and Discussion

In our KPFM measurements, we studied a HWC of the type lithium (Li)|lithium phosphate (LPO)|gold (Au) as a model system (Figure 10a). Here, lithium metal acts as the reversible electrode (RE) for both lithium ions and electrons, gold acts as lithium ion-blocking electrode (WE) in polarization direction, and LPO as MIEC. We chose LPO, as it can easily be deposited via the gas phase in vacuum and is chemically quite stable.^47^ Glassy LPO is a lithium-ion conductor with an ionic conductivity of σ­(Li^+^) = 4.9 × 10^–7^ S cm^–1^ and has an electronic conductivity of σ­(e^–^) = 1.4 × 10^–10^ S cm^–1^.^47,48^ Since the conductivities differ by 3 orders of magnitude σ­(Li^+^)/σ­(e^–^) ≈ 10^3^, we assume that glassy LPO fulfils the requirements of MIECs in HWCs.

**Figure 10:**
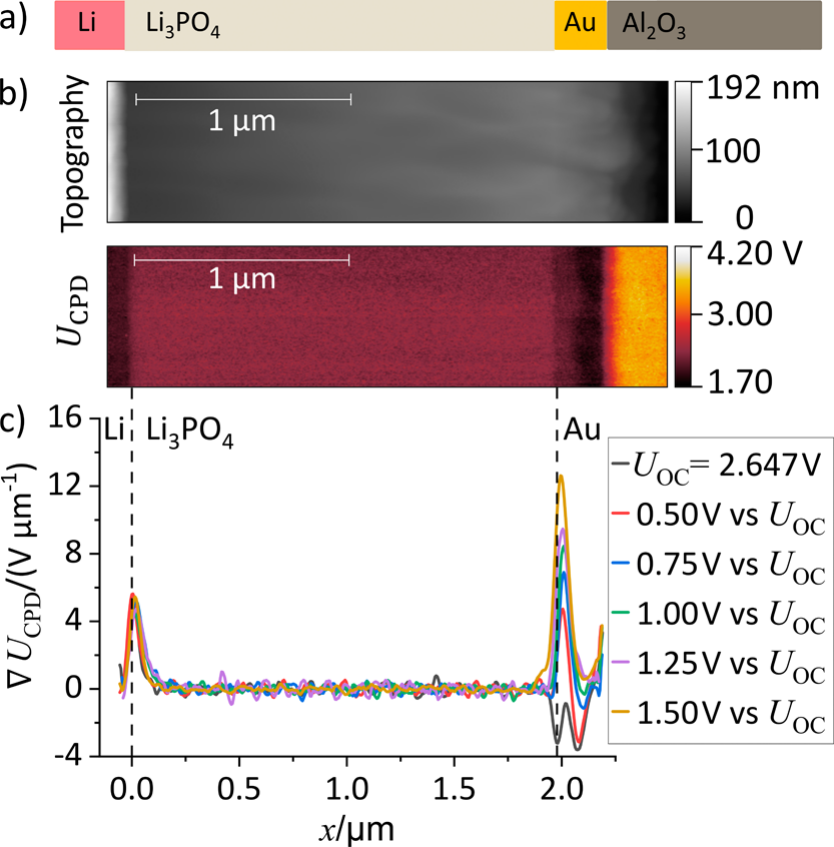
Scheme and results of a HWC in blocking direction. (a) Schematic of the HWC on an Al_2_O_3_ substrate. (b) Topography and contact potential difference (*U*_CPD_) of the polished HWC obtained at *U*_lat_ = *U*_OC_. (c) Profiles of the derivative d*U*_CPD_/*dx* = ∇*U*_CPD_ obtained from HWC cross sections for different applied polarization voltages *U*_lat_. Fluctuations in the MIEC around zero correspond to noise in the measurement.

KPFM measurements were conducted on a polished HWC cross section (Figure 10b). The open circuit voltage (*U*_OC_) of 2.647 V between WE and RE was increased by an external voltage *U*_ext_ = 0.50 V, 0.75 V, 1.00 V, 1.25 V, and 1.50 V via the connected potentiostat, respectively. Thus, the voltage *U*_lat_ between RE and WE can be written as *U*_lat_=*U*_OC_+*U*_ext_. After polarization, a current was recorded that decreased asymptotically. Once a current value falls below 10 nA (SI2), we assumed a new steady state condition and performed KPFM measurements. From the *U*_CPD_ images of the HWC cross section, *U*_CPD_ profiles (SI3) were extracted. The line profiles were then smoothed (SI4) and the derivative ∇*U*_CPD_ was calculated (Figure 10c). We indicate the positions of the interfaces to Li (RE) and Au (WE) by dashed lines. The procedure for identifying the position of these interfaces is described in SI5.

For all applied voltages *U*_lat_ the ∇*U*_CPD_ at the MIEC fluctuates around zero. As we achieve ∇φ_s_ = 0 in polarization direction, we conclude that there is indeed no surface potential gradient (∇χ_s_ = 0) in the MIEC (eq 12). The latter was already assumed by Luerßen et al. in their analysis of photoelectron microscopy along the surface of electrochemical cells.^49^ Thus, KPFM effectively measures the Galvani potential profile at the MIEC surface (∇*U*_CPD_ = ∇φ_s_). Only at the interfaces to the electrodes we found peaks in the ∇*U*_CPD_ profiles. We attribute these peaks to the presence of space charge layers, that have yet rarely been observed directly.^50^ We summarize the potential profiles acting within the HWC in Figure 11. The potential profiles are numbered from 1 to 4 for increasing *U*_lat_. The profile of the electrochemical potential of electrons varies with the applied voltage.^45^ The Galvani (φ_s_), surface (χ_s_), and Volta (Ψ_s_) potentials remain constant along the MIEC surface. At the interface toward the WE, the Galvani potential profile depends on *U*_lat_, as the extent of the space charge layer at the interface depends on the applied potential. At the working electrode, the difference between two adjacent lines of the Galvani and Volta potentials and the electrochemical potential of electrons equals the change in the applied voltage *U*_lat_ (eq 10, see SI1 for a consideration of sign).

**Figure 11:**
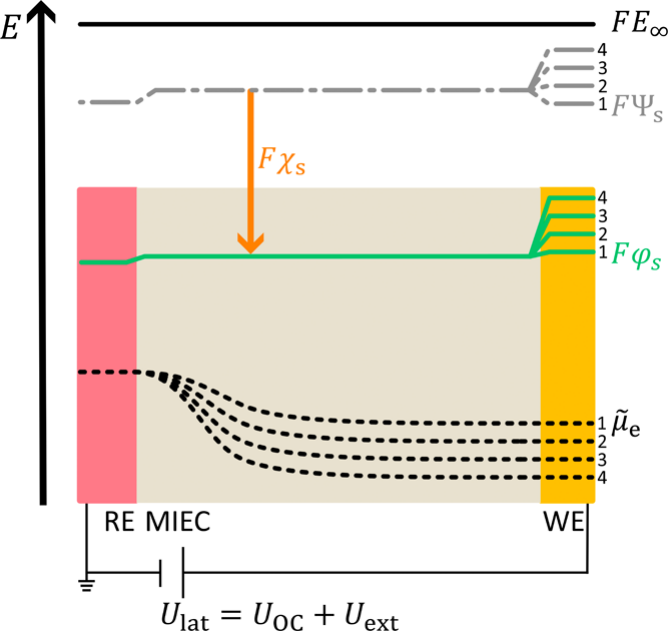
Schematic potential profiles acting in the HWC for increasing polarization voltages *U*_lat_ (1–4).

Next, we performed KPFM measurements on the same cross section, while reversing the direction of the voltage applied to the cell (Figure 12a). In this case, the cell does not act as a HWC because the ionic current is not blocked anymore. Instead, the applied voltage causes a Galvani potential gradient within the MIEC. The subsequent ionic current causes Li to be deposited on the Au electrode, leading to the formation of a Li_*x*_Au alloy (SI6).

**Figure 12:**
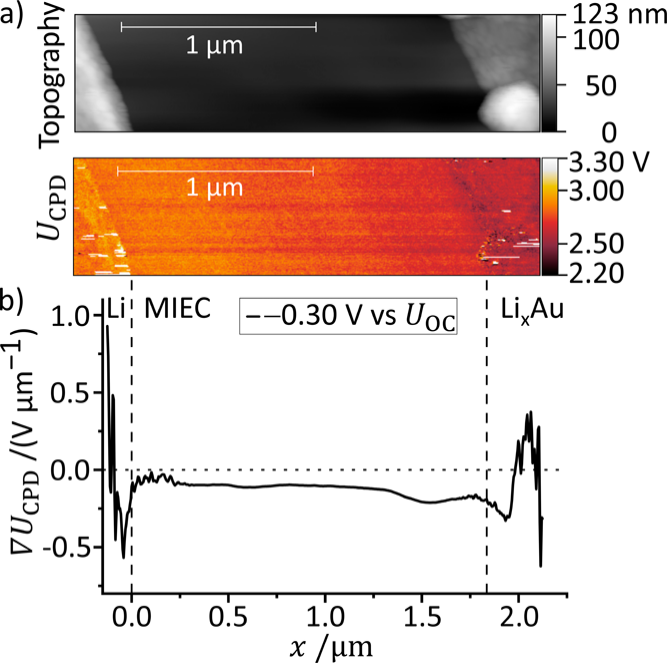
Operation of a HWC in reversible direction (lithium plating). (a) Topography and contact potential difference (*U*_CPD_) of the polished cell obtained at *U*_lat_ = *U*_OC_ + *U*_ext_ with *U*_ext_ = −0.30 V and *U*_OC_ = 0.32 V. We attribute the drift in the image to heating caused by the ionic current and formation of the Li_*x*_Au alloy. (b) Profile of the derivative d*U*_CPD_/d*x* = ∇*U*_CPD_ calculated from the *U*_CPD_ map in (a).

A lateral voltage of *U*_lat_ = *U*_OC_ + *U*_ext_ with *U*_ext_ = −0.30 V was applied between Au as the working electrode (WE) and Li as the reference electrode (RE). The *U*_CPD_ profile of the cross section was extracted and smoothed (SI7) similarly to procedure above. From the smoothed line profiles the derivative ∇*U*_CPD_ was calculated (Figure 12b). We find that the gradient ∇*U*_CPD_ is always negative at the MIEC surface.

Analogous to the HWC, we assume that the chemical potential of lithium ions remains constant in the MIEC due to their high concentration. Thus, there is no concentration gradient of lithium ions at the surface that could alter the surface potential. We conclude that the surface potential at the MIEC is constant (∇χ_s_ = 0). Thus, ∇*U*_CPD_ = ∇φ_s_, which means that the observed gradient at the MIEC corresponds to the Galvani potential gradient acting inside the MIEC. The Galvani potential gradient drives the lithium ions from the RE toward the WE. We summarized the potential profiles acting within the cell in Figure 13. The difference in the electrochemical potential of electrons ( μ̃ _e_) between the electrodes is determined by the applied voltage. The Galvani and Volta potentials have the same gradient at the MIEC due to the constant surface potential. Therefore, KPFM can be used to determine the lateral conductivity σ (eq 13), as the drop in the internal electrostatic potential over a lateral distance *l* can be measured, i.e., the Galvani potential drop Δφ.

13 $$\sigma =\frac{I}{\Delta \varphi }\cdot \frac{l}{A}$$

Here, *I* is the current flowing through the MIEC and *A* the cross-sectional area. Unlike macroscopic techniques, KPFM can spatially resolve the ionic conductivity of solid electrolytes. In this way Wang et al.^51^ quantitatively probed the ionic conductivity of LiZr_2_(PO_4_)_3_ and Li_1.05_Zr_1.95_Fe_0.05_(PO_4_)_3_ via KPFM and confirmed their results with macroscopic electrochemical impedance measurements. The authors concluded that this method could be useful for studying ionic pathways in structured solid electrolytes, e.g., via grain boundaries, to help optimize their ionic conductivity.

**Figure 13:**
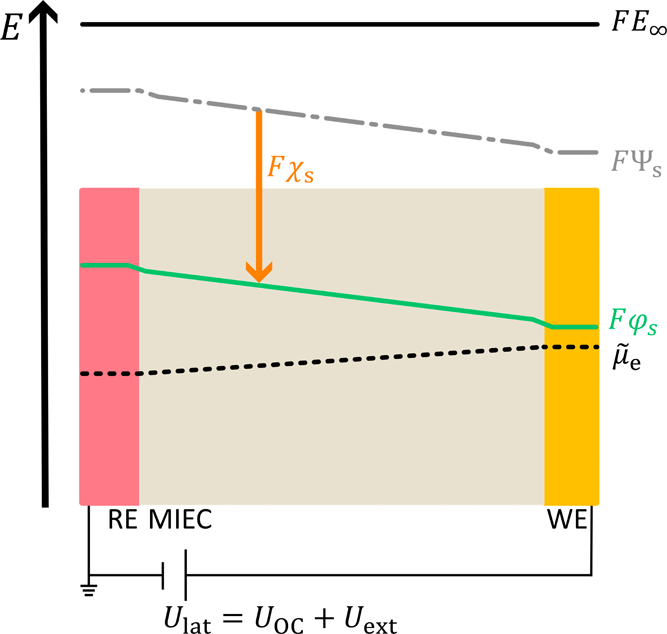
Schematic of the potential profiles acting in the cell with the applied voltage reversed to the polarization direction.

## 7 Conclusions

For pure electron and hole conductors the interpretation of the Kelvin probe force microscopy (KPFM) signal, i.e., the contact potential difference, is straightforward. In this way, KPFM can quantitatively measure the work function distribution, providing a high-resolution chemical landscape of the sample surface. However, in operating devices, the gradient in the electrochemical potential of electrons complicates the signal interpretation.

Here, we derive the KPFM signal interpretation for electrochemical cells, i.e., systems with ion-conducting materials, from basic considerations of the Volta potential and its relation to the surface potential, chemical potential of electrons, Galvani potential and work function. In particular, we discuss the effect of an external potential difference applied across a device, i.e., under operando conditions. We provide the necessary interpretation by using the nomenclature of electrochemistry to avoid conflicting terminology used by physicists and electrochemists and to make signal interpretation accessible to researchers regardless of their research background.

We emphasize that the KPFM signal depends on the Galvani *and* surface potential of the sample. Therefore, the local structure and the compositional dependence on external potentials need to be considered for a reliable signal interpretation. Only in case surface potential changes can be neglected, the KPFM signal profile corresponds to the Galvani potential profile acting inside the sample.

As a proof-of-principle for an operando measurement of electrochemical devices, we investigate a Hebb–Wagner polarization cell (HWC). Such cells are used to selectively block ion transport in a solid-state mixed ionic-electronic conductor (MIEC) and exclusively measure the electronic partial conductivity. With a HWC cell we successfully separate the contributions of the Galvani and surface potential profiles of a MIEC. We explicitly demonstrate our analysis with a HWC, thereby consolidating our general KPFM signal interpretation. In this case the inner electric (Galvani) potential profile along an operating device is measured without need of a direct mechanical contact. However, often it is not feasible to experimentally isolate Galvani and surface potential profiles in operating devices. Then numerical calculations^26^ can be useful for understanding the corresponding potential contributions in KPFM, respectively.

Our findings on MIECs contribute to a deeper understanding of KPFM measurements on MIECs. With the help of these findings, we have recently investigated the origin of lithium dendrite growth at grain boundaries of Li_6.25_Al_0.25_La_3_Zr_2_O_12_, a promising solid electrolyte candidate for all solid-state batteries.^52^ As MIECs are inevitable components in all-solid-state batteries and other electrochemical devices for energy storage and transformation, we expect a more widespread use of KPFM measurements in the future.

## 8 Experimental Section

### Hebb–Wagner Cell (HWC)

The HWC was fabricated without exposure to air.^47^ An Al_2_O_3_ single crystal with (0001) orientation was used as the substrate for the assembly of the HWC. lithium was used as the reversible electrode, lithium phosphate (LPO) as MIEC and gold as the ion-blocking electrode. The gold electrode was fabricated by DC magnetron sputtering on the Al_2_O_3_ substrate. It was performed with a power of 30 W and an argon partial pressure of 0.5 Pa. The resulting electrode had a thickness of ≈100 nm. The LPO layer was deposited by pulsed laser deposition using an ArF excimer laser (laser fluence of 1 J cm^–2^ and repetition rate of 5 Hz). The resulting LPO layer had a thickness of ≈1.5 μm. The lithium electrode was obtained by thermal evaporation. The thickness of the electrode was ≈1 μm.

### Cross Section Preparation

To reduce measurement artifacts such as topographic crosstalk,^53^ cross sections of the HWC were prepared using an argon ion milling system (IM4000Plus Hitachi) equipped with an *air-protection unit*. For Argon-ion beam polishing, a titanium mask was used to protect the sample from unintentional beam irradiation and to create a flat shadow of the ion beam. In this way, the top surface layer of the sample was polished. To reduce the polishing time, the HWC was positioned so that only the top surface was exposed to the ion beam. To avoid sample degradation due to possible dielectric breakdown, a thin glass layer was placed between the mask and the HWC. The acceleration voltage was set to ≈2 V. The discharge voltage was set to ≈1.5 kV with a discharge current of ≈450 μA. The gas flow was set to ≈0.1 cm^3^/min. The tilt angle was set to ≈90°. During polishing, the sample holder was rotated ±30° clockwise and counterclockwise at 30 reciprocations per minute. The polishing time was ≈24 h. To prevent sample degradation, transport between the measurement glovebox and the ion milling system was carried out via an air-protection chamber leaving the sample under an inert Argon-atmosphere. For a more detailed description of the experimental procedure, the reader is referred to the work of Weber.^54^ The effects of Argon ion milling on the measured *U*_CPD_ are evaluated in SI8.

### Measurement Environment

KPFM measurements were performed in a glovebox (GS Systems) with a controlled atmosphere of argon (purity: 99.9999% from Air Liquide). Traces of oxygen and water were continuously monitored: *p*(O_2_)/*p* < 3.0 ppm, *p*(H_2_O)/*p* < 1.0 ppm. A potentiostat SP-150 from BioLogic was used to apply a voltage to the sample and to monitor the current. The counter electrode (lithium electrode) was connected to ground and served as the reference electrode. A potential difference was applied to the working electrode (gold electrode).

### KPFM Measurements

KPFM measurements were performed in heterodyne frequency modulation mode^34^ using the MFP 3D from Asylum Research. For the lock-in detection required for KPFM, the lock-in amplifier (HF2 from Zurich) was used. For each measurement, the n-doped (Sb) silicon cantilever plated with a platinum–iridium alloy for electrical conductivity, designated SCM-PIT-V1, from Bruker. The nominal first resonance frequency was ω_0_ = 75 kHz. More details about the KPFM working principle are provided in SI9.

## Supplementary Material



## Footnotes

a Usually, the contact potential difference is defined as $U_{\mathrm{CPD}}=\frac{\phi_{s}-\phi_{t}}{-e}$ , where *e* is the elementary charge (1.602 × 10^–19^ C).^17^ In this case, the work function is defined per electron. Electrochemists usually define potentials per mole of particles. For consistency, we have defined the work function per mole of electrons which leads to the definition of the contact potential difference as in eq 1.

## Notes

Discussion of the sign in potential addition, measured current as a function of time in the HWC, *U*_CPD_ profile extraction, *U*_CPD_ profile smoothing, identification of interfaces of the MIEC with the electrodes, Li_*x*_Au formation, *U*_CPD_ profile smoothing of operation against polarization direction, effects of argon ion milling on the measured *U*_CPD_, and working principle of KPFM (PDF)
