# Supplementary material for: Interpretation of Kelvin Probe Force Measurements in Solid-State Electrochemical Cells
Source: ACS Appl Mater Interfaces. 2025 Oct 7;17(42):58825–36. doi: 10.1021/acsami.5c10182 (PMC12557226; doi:10.1021/acsami.5c10182)
Supplement: Supplementary file 1 [file am5c10182_si_001.pdf]

Supporting Information

Interpretation of Kelvin Probe Force Measurements in Solid-State Electrochemical Cells

Franjo Weber[1†], Chao Zhu[1†], Shigeru Kobayashi[§], Till Fuchs[‡], Taro Hitosugi[§], Jürgen Janek*[‡], Rüdiger Berger*[†]

[1] These authors contributed equally

[†] Max Planck Institute for Polymer Research, Ackermannweg 10, 55128 Mainz, Germany.

[§] Department of Chemistry, The University of Tokyo, Tokyo 113-0033, Japan

[‡] Institute of Physical Chemistry & Center for Materials Research, Justus Liebig University Giessen, Heinrich-Buff Ring 17, 35392 Giessen, Germany.

*Corresponding authors: juergen.janek@phys.chemie.uni-giessen.de; berger@mpip-mainz.mpg.de

SI1: Discussion of the sign in potential addition

Electrochemical potential

The definition of the electrochemical potential $\tilde{\mu}_\mathrm{j}$ of any charged particle j is given by Equation 4. We rewrite all relevant equations in the supporting information for clarity and renumber them. Here Equation 4 corresponds to Equation S1.

$$\tilde{\mu}_\mathrm{j} = \mu_\mathrm{j} + n_\mathrm{j} F(\chi + \Psi) \tag{S1}$$

Where n_j is the charge number. The charge number is calculated by dividing the charge of particle j by the elementary charge. For one electron the charge number is $n_\mathrm{e} = -\,e/e = -1$. The definition of the Galvani potential φ is given by Equation 3.

$$\varphi = \chi + \Psi \tag{S2}$$

We insert Equation S2 into Equation S1 and obtain:

$$\tilde{\mu}_\mathrm{j} = \mu_\mathrm{j} + n_\mathrm{j} F(\chi + \Psi) = \mu_\mathrm{j} + n_\mathrm{j} F\varphi \tag{S3}$$

Here, μ_j represents the chemical potential of the particle j. The electrostatic potentials are χ,Ψ and φ and represent the surface potential, Volta potential and Galvani potential. All electrostatic potentials are defined in such a way that the potential energy of a positive charge increases with rising electrostatic potential. To account for the effect of an electrostatic potential on particle j, the charge number n_j is present in Equation S3. Then, the sum of all vectors correspond to the electrochemical potential of the particle j. For one electron, we obtain:

$$\tilde{\mu}_\mathrm{e} = \mu_\mathrm{e} + n_\mathrm{e} F(\chi + \Psi) = \mu_\mathrm{e} - F\varphi \tag{S4}$$

Please note, that the effect of electrostatic potentials χ and Ψ acting on an electron is in the opposite direction to that of a positively charged particle. Therefore, we wrote the values of the grey and orange vectors in Figure S1 (Figure 2c) negative, *i.e.* $-F\Psi$ and $-F\chi$. The direction of the arrows is defined in such a way that their vector sum gives the electrochemical potential of electrons.

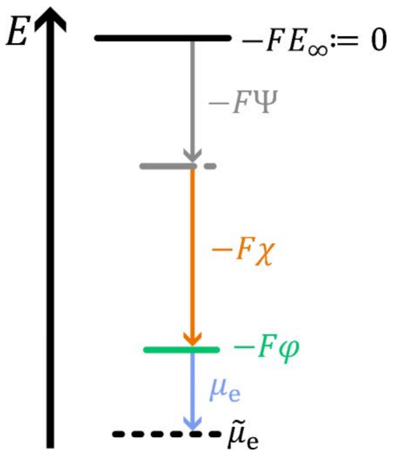

***Figure S1**: Contributions to the electrochemical potential of electrons.[1]*

Contact potential difference

The contact potential difference U_{CPD} is defined in Equation 1 of the main manuscript:

$$U_{\mathrm{CPD}} = \frac{\phi_{\mathrm{s}} - \phi_{\mathrm{t}}}{-F} \tag{S5}$$

We consider the following example: We measure an aluminium sample with work function $\phi_{\mathrm{s}} = 4\, e \cdot N_{\mathrm{A}}\mathrm{V}$ per mole of aluminum atoms. Here N_{A} is the Avogadro constant and e the elementary charge. Using $e \cdot N_{\mathrm{A}} = F$, with F being the Faraday constant, the work function can be written as $\phi_{\mathrm{s}} = 4F$ V. For the measurement we use a gold tip with a work function of $\phi_{\mathrm{t}} = 5F$ V. We insert the work functions into Equation S5 and obtain: $U_{\mathrm{CPD}} = 1$ V. For simplicity, we set $F = 1$ in the following.

Figure S2 shows the operating principle of KPFM based on Figure 3 in the manuscript. In addition, we indicated the values of work functions from the above example. Prior to the measurement, head-tip and sample-surface assemblies are separated. The electrochemical potential of electrons in the head-tip assembly is lower than in the sample-surface assembly (dotted boxes in Figure S2a). Upon electrical contact, electrons will move towards the lower electrochemical potential of electrons. As a result, electrons move from the sample-surface to the KPFM tip (Figure S2b). The tip becomes negatively charged and the surface becomes positively charged. The charging generates a Volta potential difference between the tip and the sample surface, which equals to U_{CPD} (energy diagram, Figure S2b). The magnitude of U_{CPD} is given by equation S5 and rewritten results in equation S6, which represents the vector addition (arrows pointing upwards, Figure S2b).

$$\phi_{\mathrm{t}} = FU_{\mathrm{CPD}} + \phi_{\mathrm{s}} \tag{S6}$$

KPFM operates by nullifying the Volta potential difference between tip and surface. Thus, in the example above the level $-F\Psi_{\mathrm{t}}$ (left grey arrow, Figure S2b) has to be shifted towards lower energy, as the sample is grounded and $-F\Psi_{\mathrm{s}}$ (right grey arrow, Figure S2b) stays constant. The corresponding Volta potential shift is achieved by applying a compensation voltage U_{CPD} between KPFM head and sample that alters the electrochemical potential of the tip (Figure S2c).
To nullify the Volta potential difference, the compensation voltage must be applied in the correct direction. The long line of a voltage source indicates the positive pole and the short line the negative pole. During the measurement the head is connected to the positive pole while the sample is connected to the ground (black circuitry, Figure S2c). When a positive voltage is applied, the electrostatic potential of the positive pole increases. According to the definition of the electrochemical potential of electrons in equation S4, $\tilde{\mu}_{\mathrm{e}}$ decreases for increasing electrostatic potentials. In our example we have to apply a positive voltage of $U_{\mathrm{DC}} = U_{\mathrm{CPD}} = 1$ V to the KPFM head in order to decrease $\tilde{\mu}_{\mathrm{e,t}}$. Then we obtain $-F\Psi_{\mathrm{t}} = -F\Psi_{\mathrm{s}}$.

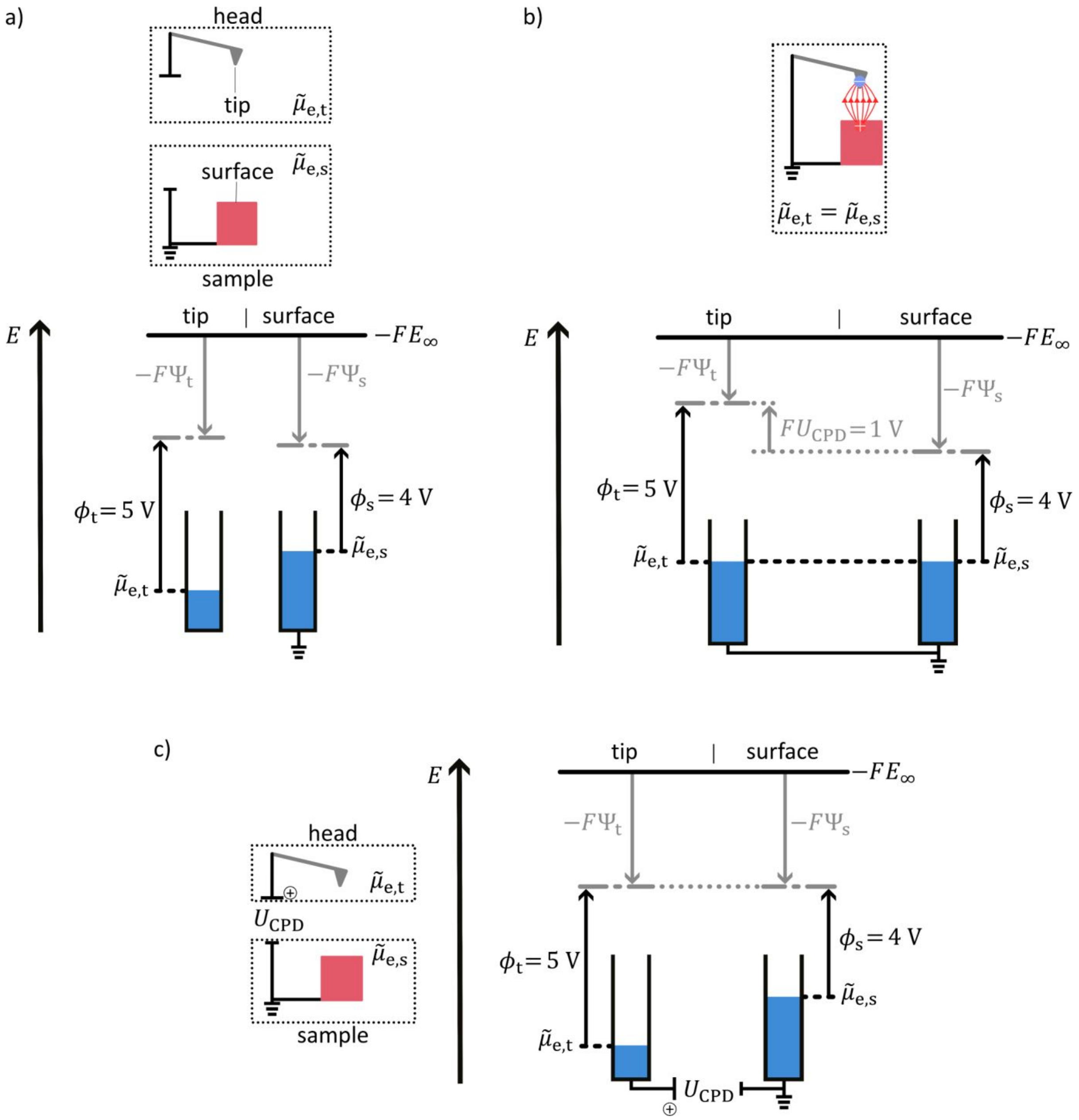

***Figure S2**: Operating principle and signal interpretation of KPFM according to Figure 3 of the manuscript. The work functions and U_{CPD} for a sample measurement described in the text are indicated, for simplicity we set $F = 1$. a) KPFM head and sample are electronically separated. b) KPFM head and sample are electronically short-circuited. c) KPFM in operation.*[1]

Contact potential difference on devices in *operando*

We consider a KPFM setup with two separated but otherwise identical metallic samples. The left sample (sample1 with surface1) is grounded. The right one (sample2 with surface2) is connected to the positive pole of the voltage source. First, we consider the measurement without an applied voltage ($U_{\mathrm{lat}} = 0$ V) between the metallic samples (Figure S3a). For the example, we again use a gold tip with a work function of $\phi_t = 5$ V and an aluminium sample with work function $\phi_s = 4$ V. The magnitude and direction of U_{CPD} between tip and surface2 is given by the vector addition of the work functions (red box, Figure S3a) identical as described in equation S5 and equation S6.
We now apply a positive voltage $U_{\mathrm{lat}} = 1$ V to the sample2 (Figure S3b). The electrochemical potential of electrons decreases for increasing electrostatic potentials. Consequently, each energy level for the

sample2 shifts downwards by 1 V. The magnitude and direction of U_{CPD} is now given by the vector addition of the work functions and the difference in the electrochemical potential of electrons (red box, Figure S3b). Equation S7 to equation S9 illustrates the vector addition based on their absolute values:

$$\phi_{\text{t}} - \Delta\tilde{\mu}_e = FU_{\text{CPD}} + \phi_{\text{s}} \tag{S7}$$

$$\phi_{\text{t}} + FU_{\text{lat}} = FU_{\text{CPD}} + \phi_{\text{s}} \tag{S8}$$

$$U_{\text{CPD}} = \frac{\phi_{\text{s}} - \phi_{\text{t}}}{-F} + U_{\text{lat}} \tag{S9}$$

Equation S9 is identical to equation 10 in the manuscript.

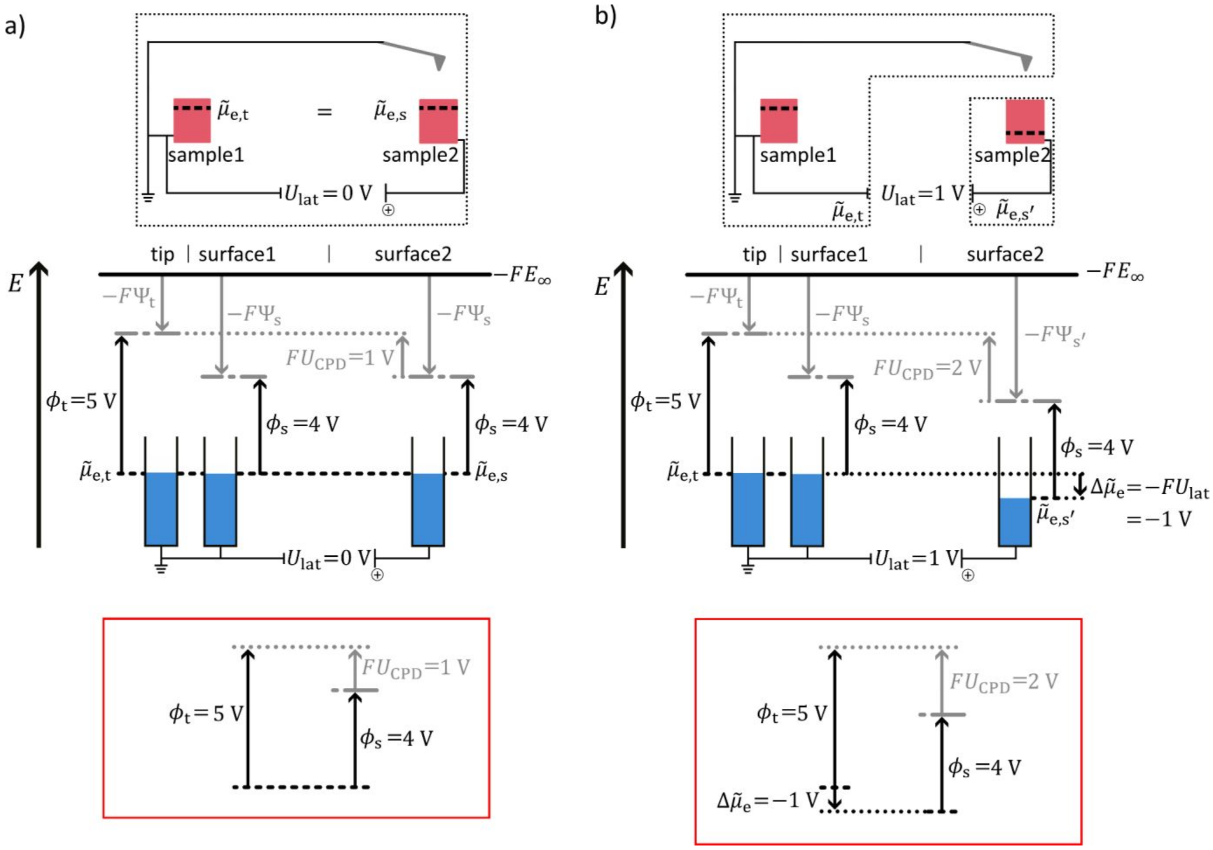

***Figure S3**: Operating principle and signal interpretation of KPFM on samples with an externally applied lateral voltage. The KPFM setup is shown in the short-circuited case. The work functions and U_{CPD} for a sample measurement described in the text are indicated, for simplicity we set $F = 1$. a) No externally applied voltage ($U_{lat} = 0\ V$). b) Positive externally applied voltage ($U_{lat} = 1\ V$).*

Contact potential difference on the working electrode of HWC

The voltage U_{lat} present between the WE and RE always represents the difference in the electrochemical potential of electrons. For a particular applied voltage we can write:

$$U_{\text{lat},1} = \mu_{\text{e,WE},1} - \mu_{\text{e,RE},2} \tag{S10}$$

By inserting the definition of the electrochemical potential of electrons into equation S10 we obtain:

$$U_{\text{lat},1} = \frac{\left(\mu_{\text{e,WE},1} - F\varphi_{\text{WE},1}\right) - \left(\mu_{\text{e,RE}} - F\varphi_{\text{RE}}\right)}{F} \tag{S11}$$

Likewise, for a higher applied lateral voltage $U_{\text{lat},2}$ we write:

$$U_{\text{lat},2} = \frac{\left(\mu_{\text{e,WE},2} - F\varphi_{\text{WE},2}\right) - \left(\mu_{\text{e,RE}} - F\varphi_{\text{RE}}\right)}{F} \tag{S12}$$

Since the WE is made up of gold, the composition remains constant upon changing the applied voltage. Thus, the chemical potential of electrons will remain constant too ($\mu_{\text{e,WE},2} = \mu_{\text{e,WE},1}$). Consequently, from subtracting equation S12 from equation S11, we obtain:

$$\begin{aligned} U_{\text{lat},2} - U_{\text{lat},1} = {} & \frac{\left(\mu_{\text{e,WE},2} - F\varphi_{\text{WE},2}\right) - \left(\mu_{\text{e,RE}} - F\varphi_{\text{RE}}\right)}{F} \\ & - \frac{\left(\mu_{\text{e,WE},1} - F\varphi_{\text{WE},1}\right) - \left(\mu_{\text{e,RE}} - F\varphi_{\text{RE}}\right)}{F} \end{aligned} \tag{S13}$$

$$U_{\text{lat},2} - U_{\text{lat},1} = -(\varphi_{\text{WE},2} - \varphi_{\text{WE},1}) \tag{S14}$$

Thus, the change in the lateral applied voltage equals the negative change in the Galvani potential at the WE (Equation S14). Furthermore, by inserting the definition of the Galvani potential, we obtain:

$$U_{\text{lat},2} - U_{\text{lat},1} = (\Psi_{\text{WE},1} + \chi_{\text{WE},1}) - (\Psi_{\text{WE},2} + \chi_{\text{WE},2}) \tag{S15}$$

Since the WE is made up of gold, the composition of the surface remains constant upon changing the applied voltage. Thus, the surface potential in equation S15 will remain constant too ($\chi_{\text{WE},2} = \chi_{\text{WE},1}$). Consequently, the change in the lateral applied voltage equals the negative change in the Volta potential at the WE:

$$U_{\text{lat},2} - U_{\text{lat},1} = -(\Psi_{\text{WE},2} - \Psi_{\text{WE},1}) \tag{S16}$$

In conclusion, the change in the electrochemical potential of electrons at the WE occurs in the opposite direction to the change of the Galvani potential and the measured Volta potential (Equation S16) via KPFM at the WE.

SI2: Measured current as a function of time in the HWC

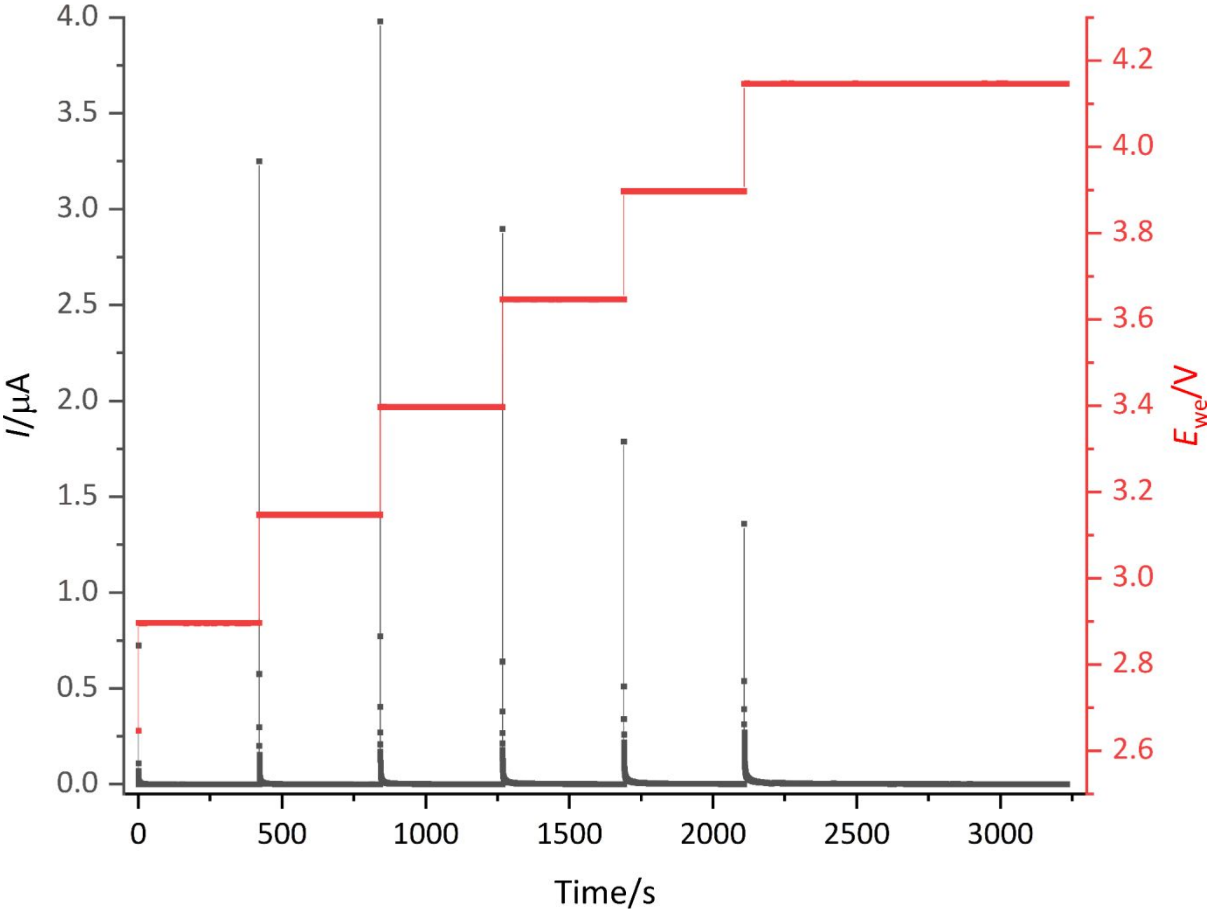

Figure S4: *Measured current I and the applied voltage U to the HWC.*

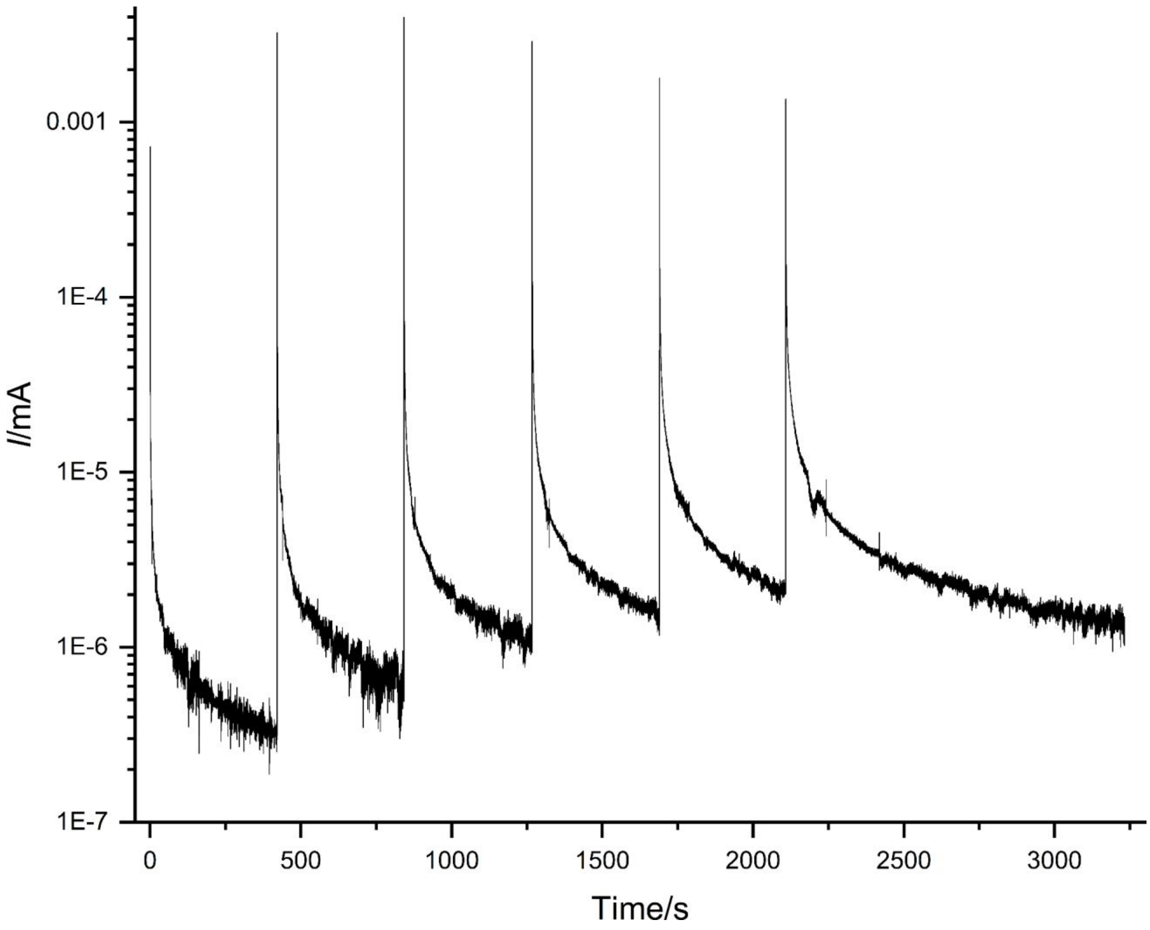

Figure S5: *Measured current I plotted on a logarithmic scale as a function of polarization time.*

SI3: U_{CPD} Profile extraction

The U_{CPD} profile at the HWC cross section was extracted from the corresponding U_{CPD} map by averaging over 40 adjacent lines. For the profile extracted at U_{OC}, the selected line profile in the U_{CPD} map is shown in Figure S6. To account for drift during subsequent measurements a reference point in the corresponding phase image was used as a guide to extract the U_{CPD} profiles at the same position. The position of the selected U_{CPD} profile in Figure S6 is shown relative to the reference point (blue dotted line and circle) in Figure S7. The procedure was repeated for each applied polarization voltage. The extracted line profiles are shown in Figure S8.

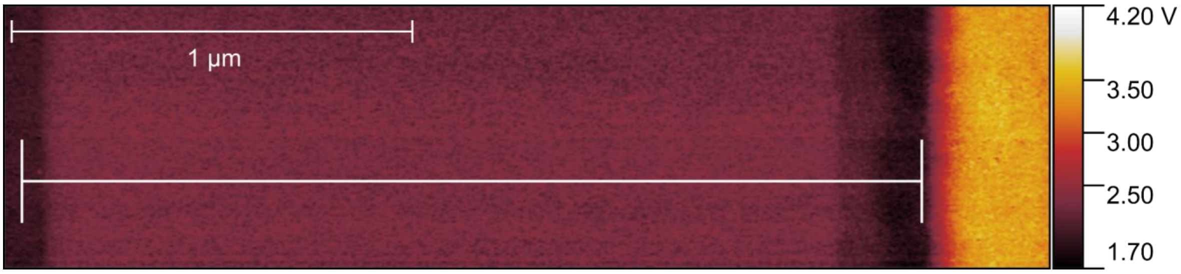

Figure S6: *U_{CPD} image of the polished cross section of the HWC at U_{OC} with selected line profile.*

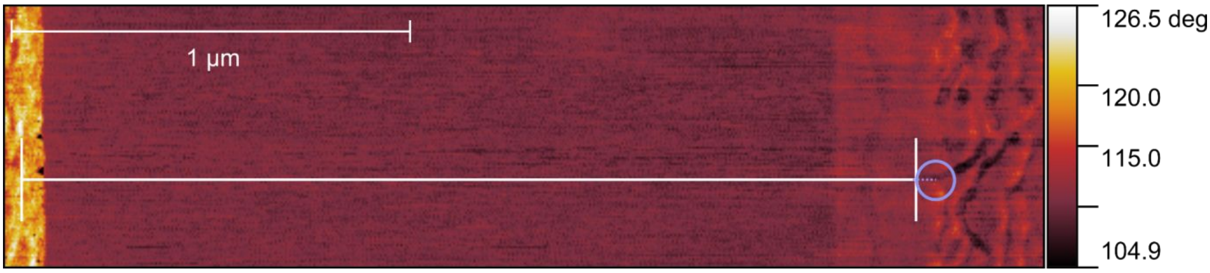

Figure S7: *Corresponding phase image of Figure S6 with the position of line profile shown relative to the used reference point.*

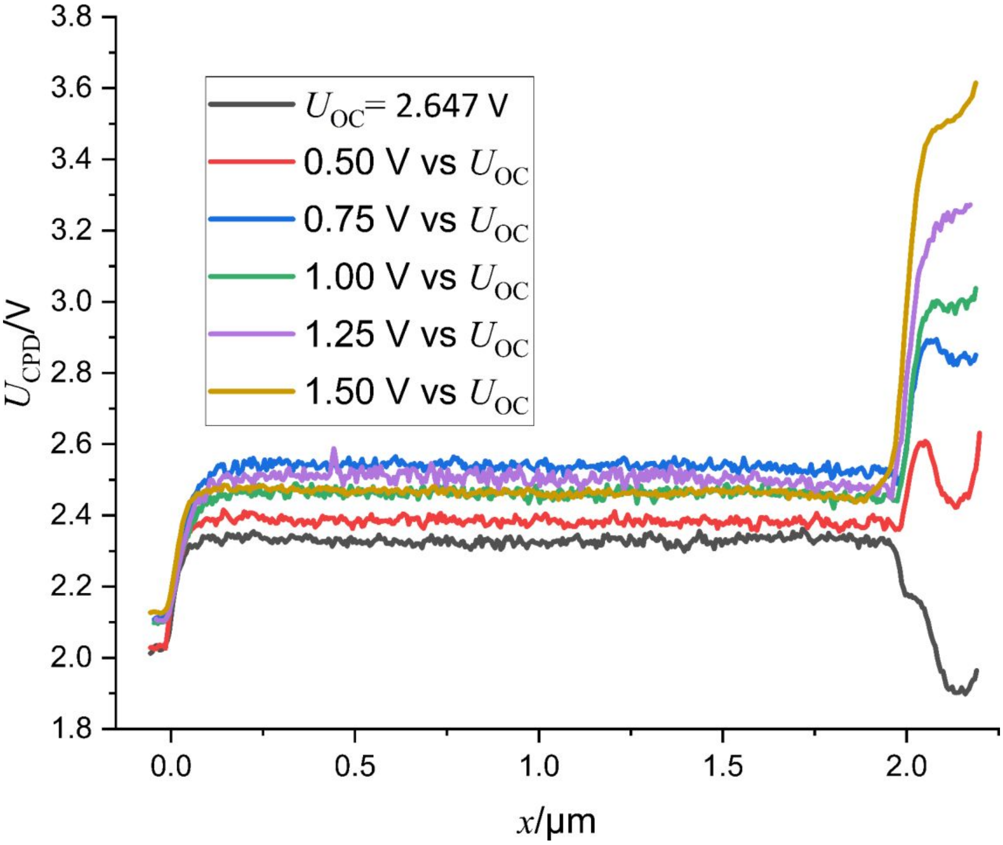

Figure S8: *Extracted U_{CPD} line profiles for each applied polarization voltage U_{lat}.*

In case of a grounded lithium electrode, $\tilde{\mu}_e$ equilibrates between this electrode and the SFM probe. This situation corresponds to the conceptual closed-circuit state (Figure 3b). Thus, KPFM measures the work function at the reversible electrode and U_{CPD} should be constant.

The Li electrode is a reversible reference electrode (Equation S17). In equilibrium, the reaction should keep the space charge layer at the interface towards the MIEC constant. Thus, the Galvani potential in the MIEC should also be constant. A constant Galvani potential in the MIEC would result in a constant U_{CPD} measured at the MIEC.

$$Li^+ + e^- \rightleftharpoons Li^0 \tag{S17}$$

We measured a constant U_{CPD} along the MIEC but the absolute value varies non-systematic by 0.3 V. We attribute these small changes to a variation in the tip Volta potential caused by deposition or erosion of material during the scanning. The Volta potential is very sensitive to surface

contamination.[2] This artifact should result in a more or less random shift of U_{CPD} that is independent of the applied voltage, which is consistent with the results in Figure S8.

SI4: U_{CPD} profile smoothing

The obtained U_{CPD} profiles (Figure S8) were smoothed (Figure S9) using the adjacent averaging method with an averaging window of 10 pixels in *OriginPro 2022*.

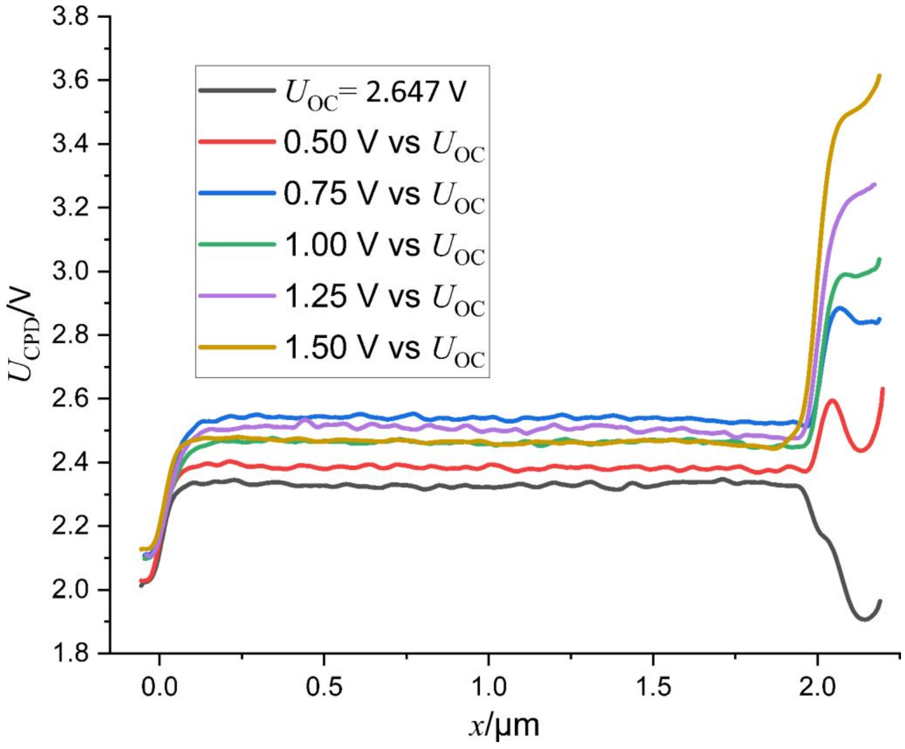

Figure S9: *Smoothed U_{CPD} profiles from Figure S8.*

SI5: Identification of interfaces of the MIEC with the electrodes

The position of the interfaces in the HWC cross section was identified with the corresponding Scanning Force Microscopy phase image, as it provides a compositional contrast[3]. Figure S10 shows an example for the data obtained at U_{OC}.

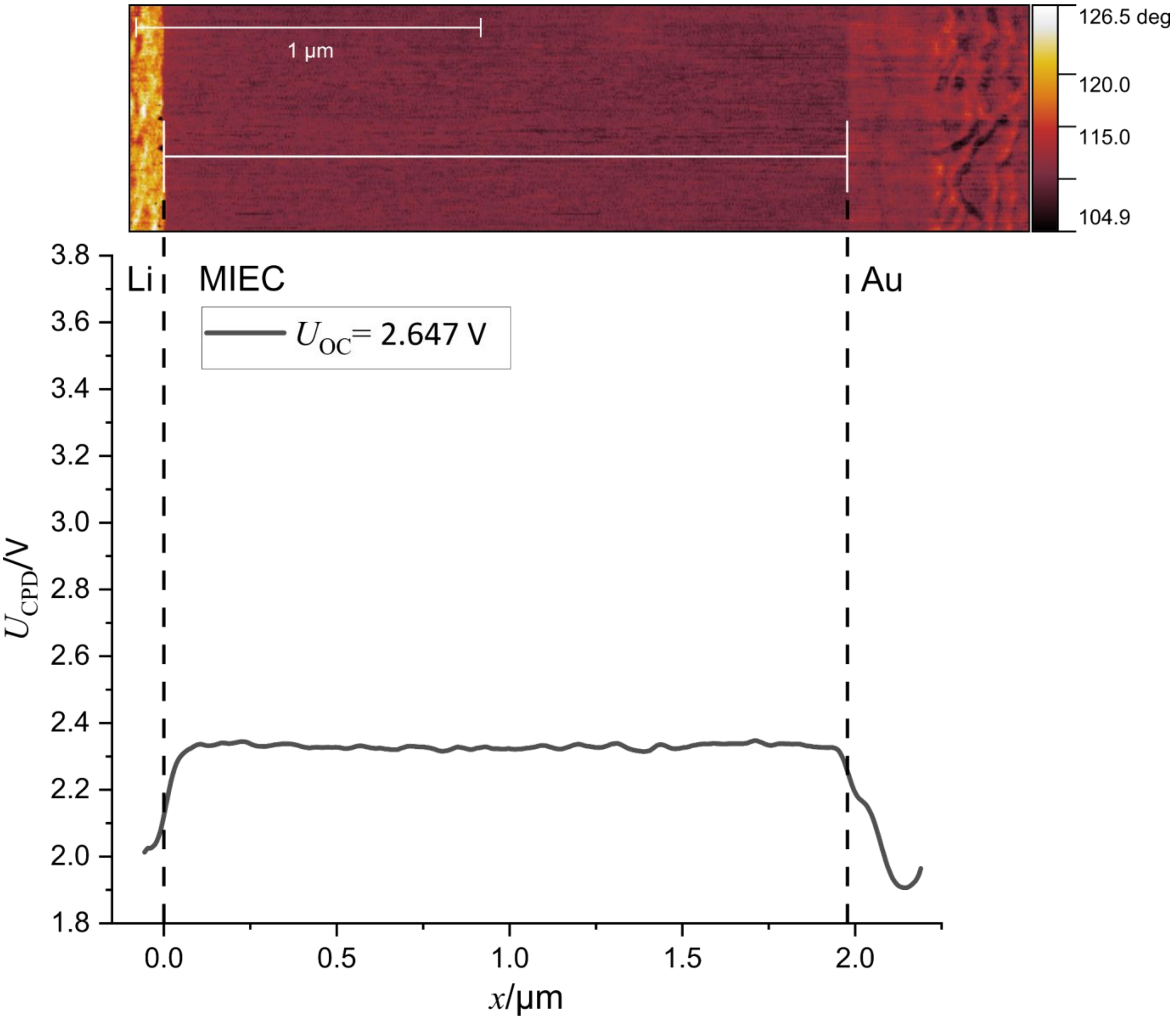

Figure S10: *Phase image (top) obtained at U_{OC} with a line selection marking the position of the interfaces between MIEC and the electrodes. Identification of the interfaces in the corresponding smoothed U_{CPD} profile (bottom).*

SI6: Li_xAu formation

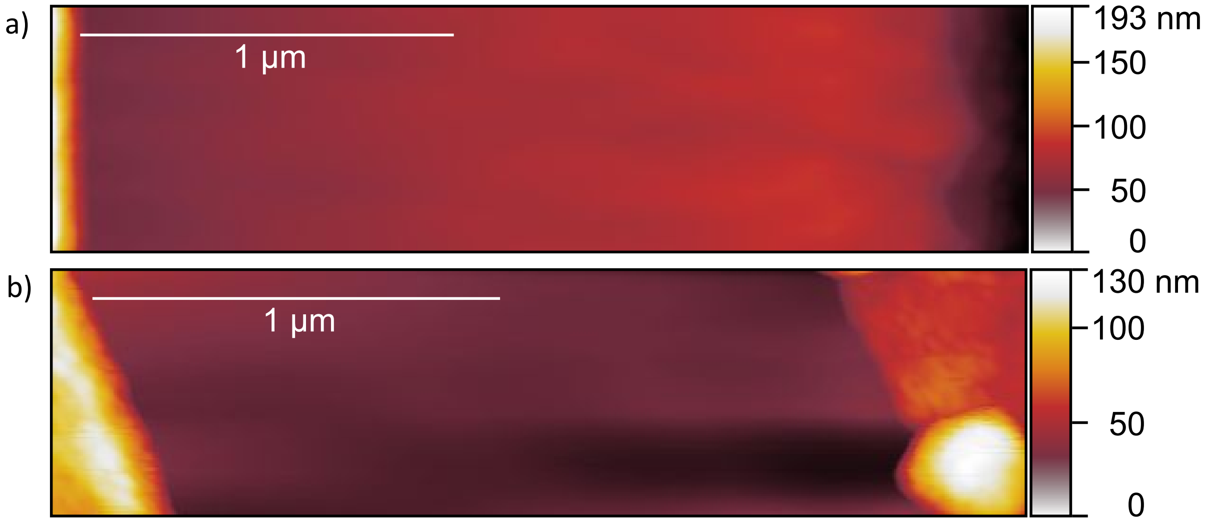

Figure S11: *Topography image of the polished cross section of the HWC at U_{OC} (a) and while applying a voltage opposite to the polarization direction (b).*

Figure S11a) shows the topography of the polished cross section of the HWC measured during polarization. Figure S11b) shows the topography of the cross section measured while a voltage opposite to the polarization direction is applied to the cell. The emerging elevation in Figure b) compared to a) on the gold electrode can be explained by the deposition of lithium onto this electrode and the formation of an alloy, namely Li_xAu.

SI7: U_{CPD} profile smoothing of operation against polarization direction

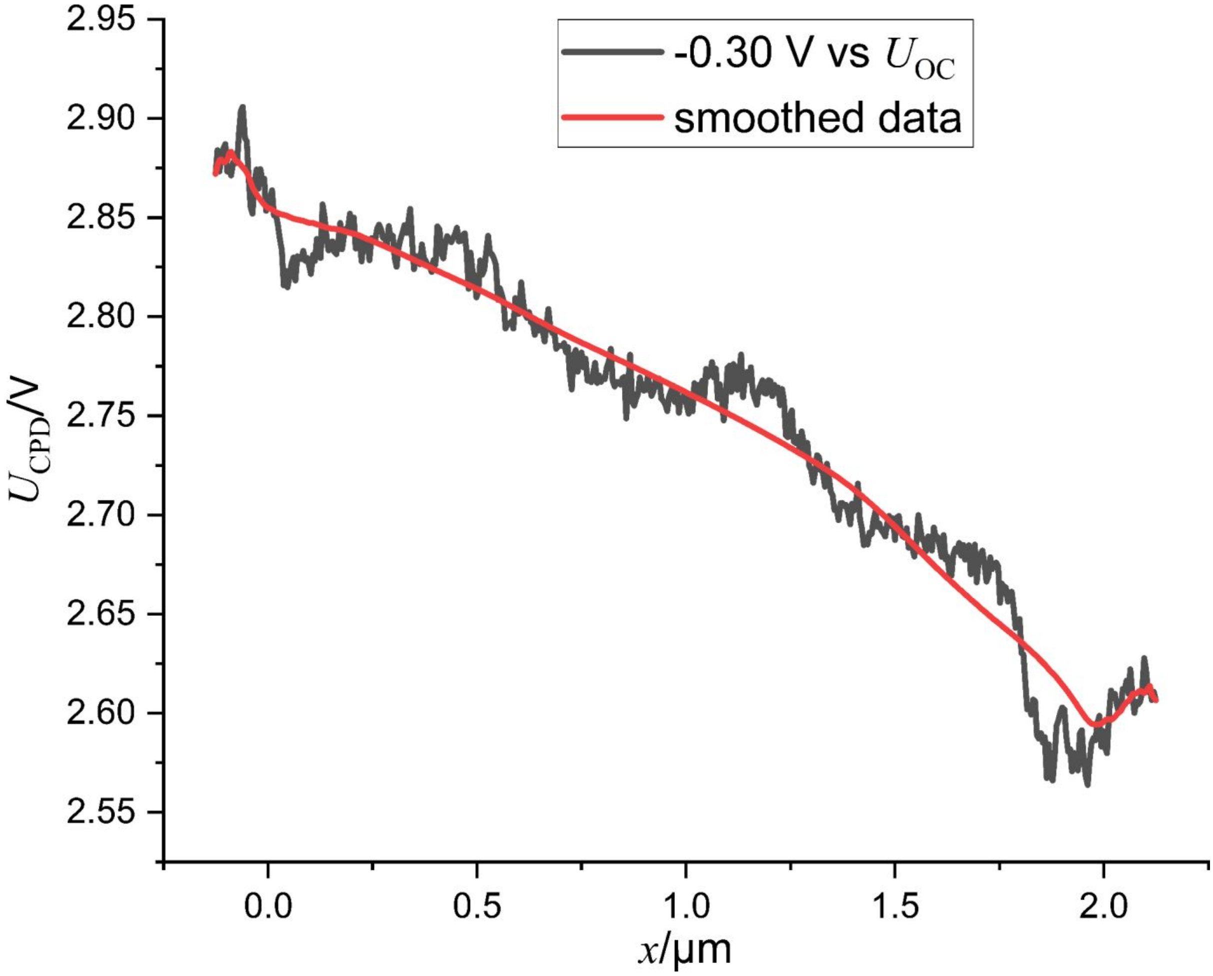

Figure S12: *Extracted U_{CPD} line profile for an applied $U_{ext} = -0.30\,V$ against U_{OC} and smoothed graph using the adjacent averaging method with an averaging window of 150 pixels. Averaging is required only to calculate the derivative. Otherwise signal noise dominates. The current in the cell was not constant during operando KPFM, possibly due to interfacial changes caused by Li_xAu formation. For this reason, we did not calculate the conductivity of the LPO layer.*

SI8: The effects of Argon ion milling on the measured U_{CPD}

In general argon is inert. Materials that are not reactive are not expected to be chemically altered by Ar-Ion milling. In a recent study C. Zhu[4] investigated the influence of Ar-Ion milling on the measured U_{CPD} on LPO in thin film batteries. For this purpose he compared pristine freshly broken LPO surfaces with polished LPO surfaces via Ar-Ion milling and measured both via KPFM. He found that the U_{CPD} on both surfaces were identical. Thus, he concluded that the Ar-Ion milling does not artificially influence the U_{CPD}. Since Au is a noble metal, we also expect that Ar-Ion milling does not artificially influence the U_{CPD} on the Au layer. In contrast, lithium is highly reactive which is why an alteration of the U_{CPD} on the Li layer cannot be ruled out. However, since our investigation focuses on the solid electrolyte LPO, our findings are independent of any possible influence of the U_{CPD} on the Li layer.

SI9: Working principle of KPFM

The electrostatic force present between the tip and the sample can be derived by considering that the tip-sample system acts as a capacitor. The normal force component can then be calculated as

$$F_{\mathrm{el}} = \frac{1}{2}\frac{\mathrm{d}C}{\mathrm{d}d}(\Delta\Psi)^2, \tag{S18}$$

with the capacitance denoted as C.[5] The KPFM measures forces indirectly by detecting the bending of the cantilever, which acts as a Hook's spring. The static force resulting from Equation S18 is in the order of pN and results in a bending close to the detection limit. To increase the sensitivity, the response is mechanically amplified by selecting frequencies at or close to resonance frequencies of the cantilever spring. In the amplitude modulation (AM) mode, an additional external voltage U_{AC} $\sin(\omega_{\mathrm{E}}t)$ is applied between probe and sample, where ω_{E} coincides with a cantilever resonance frequency ω_0. Furthermore, a DC voltage U_{DC} is applied allowing to compensate U_{CPD}.

$$U_{\mathrm{I}} = U_{\mathrm{DC}} + U_{\mathrm{AC}}\sin(\omega_{\mathrm{E}}t)$$

It follows from Equation S18, that the electrostatic force splits into the following spectral components:[5]

$$\begin{aligned} F_{\mathrm{el},\omega=0} &= \frac{1}{2}\frac{\mathrm{d}C}{\mathrm{d}d}\left((U_{\mathrm{DC}} - U_{\mathrm{CPD}})^2 + \frac{U_{\mathrm{AC}}^2}{2}\right) \\ F_{\mathrm{el},\omega_{\mathrm{E}}} &= \frac{\mathrm{d}C}{\mathrm{d}d}(U_{\mathrm{DC}} - U_{\mathrm{CPD}})U_{\mathrm{AC}}\sin(\omega_{\mathrm{E}}t) \\ F_{\mathrm{el},2\omega_{\mathrm{E}}} &= -\frac{1}{4}\frac{\mathrm{d}C}{\mathrm{d}d}U_{\mathrm{AC}}^2\cos(2\omega_{\mathrm{E}}t) \end{aligned} \tag{S19}$$

The non-static force in Equation S19 gives rise to an oscillation of the cantilever at the frequency ω_{E}. Typically, ω_{E} is selected to be the cantilever's resonance frequency. The CPD is measured by nullifying the amplitude in oscillation at ω_{E} with an electronic feedback loop varying U_{DC}. If $U_{\mathrm{DC}} = U_{\mathrm{CPD}}$ is reached, the difference in the Volta potentials of the tip and the sample surface at the frequency ω_{E} will vanish (Figure 3c). Consequently, the electrostatic force and the corresponding amplitude at the same frequency decreases to zero.[6] For imaging the CPD of an entire surface, U_{DC} is continuously adjusted as the tip is scanned, and the respective CPD value at each tip location is assigned to a pixel map. In this manuscript, we used heterodyne-frequency-mixing(FM)-KPFM[5].

The topography of the sample is imaged by mechanically exciting the cantilever with ω_{m} at its first resonant frequency $\omega_{\mathrm{m}} = \omega_0$. In addition, the cantilever is excited electronically at the frequency ω_{E}. Non-linear frequency mixing generates cantilever oscillations at sidebands at $\omega_{\mathrm{m}} \pm \omega_{\mathrm{E}}$. These sideband amplitudes are then used for the KPFM electronic feedback similar to the AM mode described above.

$$F_{\omega_{\mathrm{m}}\pm\omega_{\mathrm{E}}} = -A_m\frac{1}{2}\frac{\mathrm{d}^2C}{\mathrm{d}d^2}(U_{\mathrm{DC}} - U_{\mathrm{CPD}})U_{\mathrm{AC}}\sin[(\omega_m \pm \omega_{\mathrm{E}})t] \tag{S20}$$

Here A_m denotes the mechanical oscillation amplitude at ω_{m}. To improve the signal-to-noise ratio, the electrical excitation frequency is chosen to be the difference between the first and second mechanical resonance frequencies of the cantilever ($\omega_{\mathrm{E}} = \omega_1 - \omega_0$). We then detect the sideband in Equation S20 with a resonance amplification at the second resonance frequency ω_1. For a more detailed explanation of this technique the reader is referred to the work of Axt *et al.*.[5]

References:
1. R. A. Huggins, *Advanced Batteries*. (Springer, New York, 2009).
2. A. Alessandrini, U. Valdrè, *Philosophical Magazine Letters* **83**, 441-451 (2010).
3. J. Tamayo, R. Garcıa, *Applied Physics Letters* **73**, 2926-2928 (1998).
4. C. Zhu, Johannes Gutenberg-Universität Mainz, Mainz (2023).
5. A. Axt, I. M. Hermes, V. W. Bergmann, N. Tausendpfund, S. A. L. Weber, *Beilstein J Nanotechnol* **9**, 1809-1819 (2018).
6. J. M. R. Weaver, *Journal of Vacuum Science & Technology B: Microelectronics and Nanometer Structures* **9**, (1991).
